# Physiological and Transcriptome Analysis Reveal the Underlying Mechanism of Salicylic Acid-Alleviated Drought Stress in Kenaf (*Hibiscus cannabinus* L.)

**DOI:** 10.3390/life15020281

**Published:** 2025-02-12

**Authors:** Hui Zhang, Guofeng Xu, Samavia Mubeen, Rujian Wei, Muzammal Rehman, Shan Cao, Caijin Wang, Jiao Yue, Jiao Pan, Gang Jin, Ru Li, Tao Chen, Peng Chen

**Affiliations:** 1 Guangxi Key Laboratory of Agro-Environment and Agric-Products Safety, Key Laboratory of Crop Genetic Breeding and Germplasm Innovation, College of Agriculture, Guangxi University, Nanning 530004, China; zhanghui7363415@163.com (H.Z.); tomking2022@163.com (G.X.); samavia.mubeen@gxu.edu.cn (S.M.); wrj000119@163.com (R.W.); muzammal@gxu.edu.cn (M.R.); caoshan202276@163.com (S.C.); wangcaijin2024@163.com (C.W.); yuejiao1120@163.com (J.Y.); 15907817278@163.com (J.P.); 2 State Key Laboratory for Conservation and Utilization of Subtropical Agro-Bioresources, College of Life Science and Technology, Guangxi University, Nanning 530004, China; liruonly@163.com; 3 Guangxi Subtropical Crops Research Institute, Nanning 530001, China; jing8118@163.com (G.J.); chent3320@outlook.com (T.C.)

**Keywords:** kenaf, drought stress, salicylic acid, transcriptome, VIGS

## Abstract

Salicylic acid (SA) plays a crucial role in alleviating drought stress in plants. However, little is known about the molecular mechanisms underlying exogenous SA on the drought tolerance of kenaf. In this study, the kenaf seedlings were subjected to physiological and transcriptomic analysis under control (CK), moderate drought stress (D), and moderate drought stress with 1 mM SA (D_SA). Under drought conditions, SA significantly improved the plant biomass, leaf area, antioxidant enzyme activities (SOD, POD, and CAT), soluble sugars, starch and proline contents, and photosynthesis, while the contents of MDA, H_2_O_2_, and O_2_^−^ were significantly decreased. A total of 3430 (1118 up-regulated and 2312 down-regulated) genes were differentially expressed in group D, compared with group CK. At the same time, 92 (56 up-regulated and 36 down-regulated) genes were differentially expressed in group D_SA compared with group D. GO and KEGG analysis showed that the differentially expressed genes (DEGs) were enriched in various metabolic pathways, such as carbohydrate metabolism, lipid metabolism, and the metabolism of terpenoids and polyketides. Results showed that the genes related to the antioxidant system, sucrose and starch synthesis, osmoregulation, ABA signal regulation, and differentially expressed transcription factors, such as *AP2/ERF4* and *NF-Y1*, were involved in the increased drought tolerance of kenaf under exogenous SA. Virus-induced gene silencing (VIGS)-mediated silencing of salicylate binding protein 2 gene (*HcSABP2*) decreased the drought resistance of kenaf seedlings. Thus, the present study provides valuable insights into the regulatory mechanism of exogenous SA in alleviating drought stress in kenaf.

## 1. Introduction

Drought is one of the most important environmental factors threatening global agricultural production. Limited water resources exacerbate the impact of dry weather conditions on crop production [1]. Inadequate water supply leads to growth retardation, metabolic disorders, and reduced productivity in plants. Under drought stress, plants trigger a range of morphological, physiological, and biochemical mechanisms, including changes in leaf color, the closure of stomata, reactive oxygen species (ROS) scavenging and transcriptional activation [2]. Plants exhibit compromised morphogenesis and reduced efficiency in photosynthetic electron transport when their antioxidant defense systems fail to maintain proper redox balance, resulting from the overaccumulation of reactive oxygen species. These include superoxide anions (O_2_^−^), hydrogen peroxide (H_2_O_2_), monomorphic oxygen (^1^O_2_), and hydroxyl radicals (**·**OH) [3]. Plants contain enzymatic antioxidants (superoxide dismutase, catalase, and peroxidase) and non-enzymatic antioxidants (ascorbate and glutathione) to eliminate drought-induced peroxide accumulation [4]. At the same time, plants can accumulate various organic and inorganic substances (such as mannitol, proline, glycine, betaine, trehalose, fructose, inositol, and inorganic ions) in their cells, which are important for maintaining physiological activities during prolonged drought [5].

Kenaf (*Hibiscus cannabinus* L.) is one of the most important bast fiber crops and belongs to the Malvaceae family. It is mainly cultivated in the temperate, subtropical, and tropical areas of Asia and Africa [6]. It is characterized by drought tolerance, salt tolerance, ease of cultivation, marginal land use, high fiber yields, and a variety of industrial applications [7,8,9]. It generally grows to a height of 4 to 6 m and can reach a diameter of 2 to 3 cm in about four months [10]. Kenaf is widely cultivated in water-scarce areas because it is well adapted to the dry environment [11,12]. However, severe drought climate is now becoming more and more frequent, and drought stress is still the main cause of loss in kenaf production.

Plant hormones are essential for plants to adapt to challenging environments. For example, abscisic acid (ABA), ethylene (ET), jasmonic acid (JA), and salicylic acid (SA) are involved in the abiotic stress response of plants [13]. SA is a significant endogenous signal among other plant hormones that helps increase a plant’s resistance to heavy metal stress, dehydration, and salt damage [14]. Recent studies have shown that SA contributes significantly to plants’ capacity to withstand drought, and a certain amount of SA application under drought stress can alleviate the damage caused to plants by drought stress [15,16]. It is well known that SA is crucial in controlling the REDOX balance of cell membranes, which increases the activity of antioxidant enzymes and counteracts the harmful effects of ROS brought on by oxidative stress [17,18,19]. Additionally, SA improved plant performance in response to abiotic stressors such as heat, ozone, UV-B, heavy metals, permeability, and salt stress [20].

In the context of plant stress responses, SA functions prominently as a hormone that mitigates the effects of water scarcity [21,22,23]. However, many factors determine the right effect of SA to alleviate drought damage, such as SA concentration, plant genotype, developmental stage, organs, and so on [24,25,26,27]. Till now, the mechanism of the plant’s response to exogenous SA under the abiotic stress condition is still not clearly revealed, particularly in fiber crops. Research is needed to investigate how SA affects abiotic tolerance, and the molecular response associated with drought stress induced by SA.

We hypothesized that exogenous SA application can improve kenaf growth performance by reducing oxidative stress under drought conditions. To this end, in the present study, we explore the mechanism of SA in alleviating drought stress on kenaf growth, and the physiological and transcriptome responses of kenaf were comprehensively analyzed under drought stress conditions and exogenous SA treatment. In addition, critical DEGs and metabolic pathways were characterized to respond to kenaf drought stress. The present study will provide a foundation for dissecting the molecular mechanism of SA-alleviated drought stress in plants.

## 2. Materials and Methods

### 2.1. Materials and Experimental Design

The experimental protocol commenced with the selection of healthy seeds from kenaf cultivar CP085. Surface sterilization was achieved through sequential immersion in 70% ethanol (2 min) and 1.5% sodium hypochlorite solution (10 min), with intermediate and final rinsing using deionized water (three times). Germination conditions were maintained in sterile Petri dishes prepared with double-layered filter paper moistened with deionized water. Two days after germination, seedlings were transferred to pots (14 cm high and 15.5 cm in diameter) containing 500 g of dry soil as a growing medium and placed in a TP-R series plant growth chamber (Zhejiang Topcloud Agriculture Technology Co., Ltd., Hangzhou, Zhejiang, China) for 12 h/12 h (light/dark) cycles under optimal growth conditions of 25 °C/30 °C and 300 μmol m^−2^**·**s^−1^ light intensity.

Based on the preliminary experiment, moderate drought stress (D) and 1 mM SA (D_SA) were selected for the present study. The SA concentration was selected according to the growth status of kenaf seedlings during SA pretreatment (Figure S1). After 3 weeks, uniformly growing seedlings with 7–9 true leaves were divided into a control group (80% of field water capacity, CK), a moderate drought treatment group (45% of field water capacity, D), and a drought and SA treatment group (1 mM SA was sprayed on both sides of kenaf leaves at 9:00 am and 6:00 pm daily under moderate drought condition, D_SA; the other two groups were sprayed with the same volume of water as the control, and the spray effect was that both sides of the leaves were evenly covered by the solution). Spray of SA was started 3 days before drought treatment and continued to the second day of drought treatment. The gravimetric determination of soil water content was performed according to the standard protocol, where SWC% = [(FW − DW)/DW] × 100%. FW corresponds to the mass of field-moist soil samples collected from experimental pots, whereas DW represents the constant mass achieved after drying the samples at 85 ± 2 °C for 72 h in a DHG series hot air oven (Shanghai Jinghong Laboratory Equipment Co., Ltd., Shanghai, China). For each experimental condition, three biological replicates were established, with each replicate comprising a group of 10 seedlings.

### 2.2. Sampling, Harvesting, and Data Collection

Kenaf seed CP085 was obtained from the Key Laboratory of Crop Genetic Breeding and Germplasm Innovation, College of Agriculture, Guangxi University, and planted in the plant growth chamber, with the temperature maintained between 25–30 °C. Kenaf was treated with treatment materials in the third week of planting, transcriptome materials were collected in the third week, and materials needed for physiological experiments were collected in the fourth week. Physiological analysis and data sorting were carried out within one week after the materials were collected.

### 2.3. Plant Growth and Biomass

Morphometric analysis was performed to quantify seedling growth parameters, where vertical growth was measured using a standard ruler (cm), while stem cross-sectional dimensions were determined with a precision digital caliper (mm). The fresh weight of the whole plant was measured using an electronic balance. Leaf area was calculated using length-width product factor analysis [28].

### 2.4. Determination of Reactive Oxygen Species, Lipid Peroxidation, and Antioxidant Enzyme Activities

Using hydroxylamine oxidation, the contents of H_2_O_2_ and O_2_^−^ were determined [29]. The presence of superoxide radicals (O_2_^−^) was identified by nitroblue tetrazolium (NBT) staining, while hydrogen peroxide (H_2_O_2_) was histochemically detected using the 3,3’-diaminobenzidine (DAB) method [30]. The level of lipid peroxidation was assessed by measuring the concentration of malondialdehyde (MDA). With a small modification, the cold trichloroacetic acid (TCA) technique was used to extract the MDA content [31]. The activities of key antioxidant enzymes, namely superoxide dismutase (SOD), peroxidase (POD), and catalase (CAT), were quantified using standardized biochemical assays as previously reported [32]. Briefly, following initial centrifugation, the clarified supernatant was resuspended in an extraction buffer comprising 50 mmol·L^−1^ potassium phosphate (pH 7.0), 0.4 mmol·L^−1^ EDTA, 5 mmol·L^−1^ ascorbate, and 2% PVP. Subsequent centrifugation at 12,000 rpm (20 min) yielded the final supernatant used for antioxidant enzyme assays. The contents of AsA and GSH were determined according to the methods described by Hodges [33] and Li [34], respectively.

### 2.5. Determination of Proline, Soluble Sugar, Starch, and SA Content

Proline content was measured using the ninhydrin method [35]. Briefly, 0.5 g of fresh leaf tissue was ground in 5 mL of a 3% sulfosalicylic acid solution. After heating the homogenate in a boiling water bath for 10 min, it was placed on ice to cool. Then, 2 mL of the liquid was extracted and mixed with 2 mL of glacial acetic acid and 2 mL of 2.5% acid ninhydrin reagent. The mixture was then heated in a boiling water bath for 30 min. The upper organic phase layer was extracted with 4 mL of toluene after cooling, and the absorbance was measured at 520 nm. The anthrone method was used to determine the total soluble sugars and starch in leaves [36]. The levels of salicylic acid were measured using an ELISA kit purchased from Shanghai Coaibo Company (Shanghai, China).

### 2.6. Determination of Relative Electrical Conductivity (REC)

For the determination of relative electrical conductivity, leaves were washed with deionized water and filter paper was used to absorb water from the surface of the leaves. Following the addition of 10 mL deionized water to a weighing bottle holding plant leaves, air removal was achieved through 10 min of vacuum exposure in a drying oven coupled with pump-assisted evacuation. After 60 min of continuous agitation on a mechanical shaker, baseline electrolytic conductivity (R1) was recorded using a LeiCi DDS-307 analyzer (Shanghai, China). Subsequent thermal treatment involved immersing the sample-containing vessel in boiling deionized water (100 °C) for 10 min to terminate biological activity in plant tissues. The weighing bottles were cooled using running water shaken well and then measured. Electrical conductivity (R2) was measured using Orion Versa Star Pro Conductivity Bench Meter (Thermo Fisher Scientific, Inc., Waltham, MA, USA) and the relative conductivity was measured according to the formula (R1/R2) × 100 [37].

### 2.7. Determination of Gaseous Exchange and Chlorophyll Content

The Li-6800xt portable photosynthesis system (LI-COR; Lincoln, NE, USA) was used to measure net photosynthetic rate, stomatal conductance, intercellular carbon dioxide, and transpiration rate of primary leaves in the treatment and control groups. The chlorophyll content of the second leaf from the top was measured according to Liu et al. [38]. The chlorophyll extraction protocol involved macerating 0.2 g of fresh leaf material in 10 mL of ethanol–acetone solvent mixture (1:2 *v*/*v*). After transferring the extract to 15 mL centrifuge tubes, samples were incubated in darkness at room temperature for 24 h to ensure complete pigment extraction, as indicated by tissue bleaching. The chlorophyll content was subsequently quantified using the following spectrophotometric equation: Total chlorophyll (mg/g FW) = (20.2 × A645 + 8.02 × A663) × V/W, where D 663 and D 645 are the wavelengths corresponding to optical density values, V is the volume of extracted liquid, and W is the weight of fresh leaves.

### 2.8. RNA Extraction

Total RNA isolation from leaf samples was conducted using the TRIzol^®^-based extraction method (Invitrogen, Carlsbad, CA, USA). RNA purity and concentration were evaluated using a NanoDrop ND-2000 spectrophotometer, while RNA integrity was verified through microfluidic capillary electrophoresis (Agilent 2100 Bioanalyzer, Agilent Technologies, Santa Clara, CA, USA). High-quality RNA samples meeting stringent quality thresholds (A260/A280: 1.8-2.2; A260/A230 ≥ 2.0; RNA integrity number ≥ 6.5; 28S:18S rRNA ratio ≥ 1.0) with a minimum quantity of 1 μg were processed for subsequent molecular analyses.

### 2.9. cDNA Library Construction, RNA Sequencing, and Reads Mapping

Library preparation for RNA sequencing was executed according to the TruSeq Stranded mRNA LT protocol (Illumina, San Diego, CA, USA). Starting with 1 μg of DNase-treated RNA, cDNA fragments averaging 300 bp were size-selected via 2% agarose gel electrophoresis (Certified Low Range Ultra Agarose, Bio-Rad, Hercules, CA, USA). Final library amplification (15 cycles) was carried out using Phusion^®^ High-Fidelity DNA Polymerase (New England Biolabs, Inc., Ipswich, MA, USA) with the following thermal profile: 98 °C/30 s; 15 × (98 °C/10 s, 65 °C/30 s, 72 °C/30 s); 72 °C/5 min. Following quantification using TBS380, the RNA sequencing libraries were subjected to sequencing on the Illumina HiSeq X Ten or NovaSeq 6000 platform, with a paired-end read length of 150 bp. Initial processing of the raw sequencing data involved trimming and quality assessment, utilizing the default settings of SeqPrep (https://github.com/jstjohn/SeqPrep) and Sickle (https://github.com/najoshi/sickle) tools (accessed on 14 January 2022). Afterward, the high-quality reads were mapped to the reference genome using the HISA T2 software (http://ccb.jhu.edu/software/hisat2/index.shtml) (accessed on 18 January 2022), employing an orientation-aware alignment strategy [39]. The reference-based transcriptome assembly was conducted using StringTie (https://ccb.jhu.edu/software/stringtie/index.shtml?t=example) (accessed on 23 January 2022) with default parameters, integrating aligned reads (BAM files) from each biological replicate to generate consensus transcript models [40].

### 2.10. Differential Expression Analysis and Functional Enrichment

To reveal the response of gene expression profiles to drought stress and the effects of SA treatment on kenaf seedlings, plant samples exposed to 45% soil water content, 1 mM SA-treated drought and a control group were selected for cDNA library construction and transcriptome analysis. The experiment of the transcriptome data by Majorbio (https://www.majorbio.com/web/www/index) (accessed on 25 January 2022) assembly sequencing. To be specific, built a dds matrix using countData data to filter low-quality low-count data (count > 1). We used DESeq data standardization for differential expression analysis. The Foldchange value and *p* value after FDR correction were selected, and the genes with a padj value (*p* value after multiple verification and correction) less than 0.05 and |log2 Fold Change| greater than 2 were selected as differential gene sets. Functional characterization of DEGs was performed through over-representation analysis using GO (biological process, molecular function, cellular component) and KEGG pathway databases. Enrichment significance was assessed through hypergeometric testing with Bonferroni multiple testing correction (adjusted *p* ≤ 0.05) against the whole genome background. The functional annotation pipeline comprised two phases: (1) GO term enrichment analysis using Goatools (https://github.com/tanghaibao/Goatools) with a significance threshold of adjusted *p* < 0.05, and (2) KEGG pathway mapping through KOBAS (http://kobas.cbi.pku.edu.cn/home.do) (accessed on 28 January 2022) [41], employing the whole annotated transcriptome as background reference.

### 2.11. Quantitative Real Time Reverse Transcription PCR (qRT PCR) Analysis

To authenticate the transcriptome data, a selection of 12 differentially expressed genes (DEGs) associated with diverse biological functions was made at random. These genes were then subjected to validation using quantitative reverse transcription polymerase chain reaction (qRT-PCR). The qRT-PCR experiments were conducted using a Bio-Rad CFX96 instrument (Bio-Rad Laboratories) with SYBR qPCR Master Mix from Vazyme Biotechnology Ltd. (Nanjing, China) [42]. Each PCR reaction mixture had a total volume of 20 µL, which consisted of 10 µL of qPCR Master Mix, 0.4 µL of forward primer, 0.4 µL of reverse primer, 1 µL of cDNA template, and 8.2 µL of ddH₂O. The thermal cycling protocol included an initial denaturation at 95 °C for 30 s, followed by 40 cycles of 95 °C for 10 s and 60 °C for 30 s [43]. The relative gene expression levels were determined using the 2^–ΔΔCT^ method. The primer sequences employed for the quantitative RT-PCR analysis are provided in Supplementary Table S7.

### 2.12. Virus-Induced Differentially Expressed Gene (DEG) Silencing (VIGS) Analysis

The virus-induced gene silencing method was used to analyze the functional DEG *HcSABP2*. Using the silence prediction online software SGN-VIGS (https://vigs.solgenomics.net/) (accessed on 9 August 2022), we predicted the functional silence region of the *HcSABP2* gene. Gene-specific primers (Table S7) were designed using primer premier 5.0, and the *HcSABP2* fragment was amplified using kenaf leaf cDNA as a template. The purified fragment of the *HcSABP2* gene was inserted into the pTRV2 vector, which is derived from the tobacco rattle virus (TRV), to create the recombinant vector pTRV2-*HcSABP2*. Subsequently, the purified pTRV1 (helper plasmid), pTRV2 (empty plasmid), and the recombinant vector pTRV2-*HcSABP2* were introduced into the Agrobacterium GV3101 strain (Biomed, Beijing, China). These strains were infiltrated into kenaf leaves according to a previously published protocol [44]. When the first true leaf appeared, randomly selected seedlings were injected with approximately 1 mL of pTRV2-*HcSABP2* Agrobacterium, while seedlings infiltrated with empty vector pTRV2 were used as negative control. Two weeks after inoculation, the newly emerged third true leaf was randomly selected, and qRT-PCR was performed using *HcSABP2*-specific primers to determine the silencing effect. Following 2 weeks of VIGS (virus-induced gene silencing), wild-type plants, plants transformed with pTRV2, and plants silenced with pTRV2-*HcSABP2* were subjected to drought stress treatment. This involved watering the plants with a 1/4 Hoagland solution supplemented with 15% PEG6000, and the solution was refreshed every 2 days. After 7 days of drought stress treatment, the phenotypes of plants in each treatment were photographed and the agronomic and physiological indicators were measured.

### 2.13. Statistical Analysis

The data were analyzed using SPSS 20.0 software, employing one-way analysis of variance (ANOVA) to assess differences among groups. Post-hoc comparisons were conducted using Duncan’s multiple range test, with significance set at *p* ≤ 0.05. The results are expressed as the mean ± standard deviation (SD) based on three independent replicates.

## 3. Results

### 3.1. SA Improved the Tolerance of Kenaf Seedlings to Drought Stress

**Figure 1:**
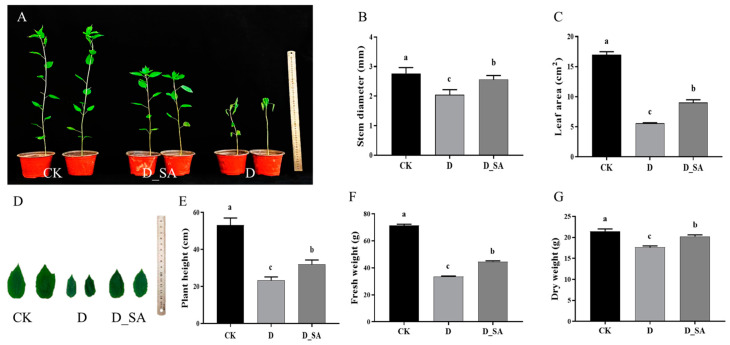
Effects of salicylic acid on (**A**) growth status of kenaf seedlings; the ruler used in the figure has a maximum measuring range of 50 cm; (**B**) stem diameter; (**C**) leaf area; (**D**) growth status of kenaf seedling leaves; the ruler used in the figure has a maximum measuring range of 15 cm; (**E**) plant height; (**F**) fresh weight; and (**G**) dry weight of kenaf seedlings grown in drought conditions. CK (control), D (moderate drought), and D_SA (1 mM SA under moderate drought stress). While the data order of (**A**–**G**) is different, the content is the same. Values are means ± SD (n = 3). Bars indicate SD. Different letters indicate significant differences in Duncan’s test at *p* ≤ 0.05.

After 7 days of treatment, seedlings in the control group grew normally, while seedlings in the moderate drought treatment group withered severely. As shown in Figure 1, kenaf seedlings under drought stress showed symptoms, such as reduced plant height, leaf shedding, curling, and wilting. Further statistical analysis showed that stem diameter, leaf area, plant height, fresh weight, and dry weight significantly decreased in the drought-treated group (D) compared with the control group (CK) by 24.51%, 67.12%, 56.04%, 52.75%, and 17.86%, respectively. In the D_SA group, stem diameter, leaf area, plant height, fresh weight, and dry weight fell by 5.33%, 46.89%, 39.83%, 37.38%, and 5.9%, respectively, compared with CK. The difference in leaf area was the most significant (Figure 1). These results indicated that exogenous SA can alleviate drought stress to some extent.

### 3.2. SA Reduced ROS Production Under Drought Stress

**Figure 2:**
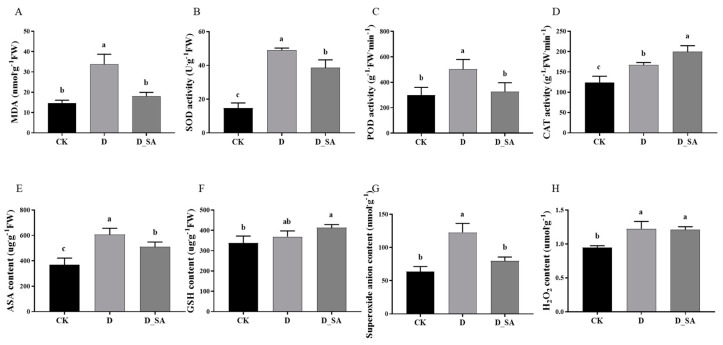
Effects of salicylic acid on (**A**) malondialdehyde (MDA) content, (**B**) superoxide dismutase (SOD) activity, (**C**) peroxidase (POD) activity, (**D**) catalase (CAT) activity, (**E**) AsA content, (**F**) glutathione (GSH) content, (**G**) superoxide anion content, and (**H**) H_2_O_2_ content of kenaf seedlings grown in drought conditions. CK (control), D (moderate drought), and D_SA (1 mM SA under moderate drought stress). Values are means ± SD (n = 3). Bars indicate SD. Different letters indicate significant differences in Duncan’s test at *p* ≤ 0.05.

Malondialdehyde (MDA) content was dramatically increased under drought stress compared with the CK group, which increased by 129.53%, indicating that the kenaf membrane could be seriously damaged by drought treatment. However, compared with the D group, the MDA content in the D_SA group dramatically decreased by 85.38%, indicating that the exogenous SA could alleviate the damage of the kenaf cell membrane caused by drought stress. Nonetheless, there was no discernible variation in MDA content between the CK group and the D_SA group (Figure 2A).

After drought treatment, the SOD activity of kenaf seedlings increased drastically, and the SOD activity of group D was 232.59% higher than that of group CK. However, SOD activity in the D_SA group was significantly increased by 27.20% compared with the CK group (Figure 2B). Similarly, under drought stress, POD activity in group D was significantly increased by 67.77% compared with the CK group. However, POD activity in the D_SA group did not change significantly compared with the CK group (Figure 2C). Furthermore, under drought stress, CAT activity in the D group dramatically increased by 34.67% as compared to the CK group. When compared to the CK group, the CAT activity in the D_SA group considerably increased by 60.72% (Figure 2D).

Compared with the CK group, AsA content in the leaves of kenaf seedlings in group D was significantly increased by 64.93%. At the same time, the AsA content in the D_SA group dramatically increased by 38.87% in comparison to the CK group. In the D group, the content of GSH in leaves of kenaf seedlings increased by 9.16% compared to group CK. GSH content was nevertheless 22.12% greater in the D_SA group compared to the CK group, which showed a significant difference (Figure 2E,F).

**Figure 3:**
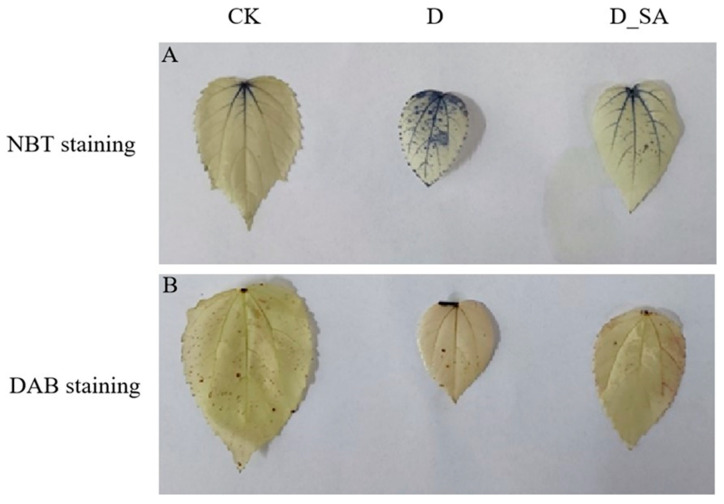
NBT and DAB staining. (**A**): NBT staining to detect O_2_^−^ content. (**B**): DAB staining to detect H_2_O_2_ content.

Reactive oxygen species (ROS) mainly include H_2_O_2_, O_2_^−^, and **·**OH. In group D, O_2_^−^ content was increased by 93.08% in kenaf seedlings, compared to group CK. But there was little difference in O_2_^−^ content between the CK group and the D_SA group (Figure 2G). The H_2_O_2_ content of kenaf seedlings was also significantly increased by 29.08% in group D compared to group CK. There was no discernible difference between the D_SA group and the CK group in terms of the H_2_O_2_ content, which was comparable to that of the D group (Figure 2H). The distribution of H_2_O_2_ and O_2_^−^ in the leaves of kenaf seedlings is also shown in Figure 3. The NBT staining area of the salicylic acid treatment group was obviously between the control group and the drought treatment group; however, there was no significant difference in DAB staining area among the three treatment groups.

### 3.3. SA Alleviates Drought Stress by Regulating the Accumulation of Osmotic Substances

**Figure 4:**
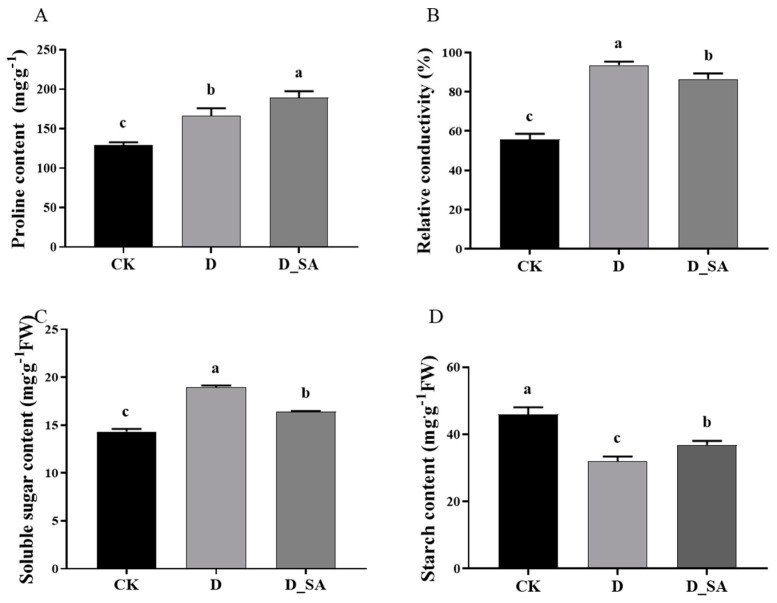
Effects of salicylic acid on (**A**) proline content, (**B**) relative conductivity, (**C**) soluble sugar content, and (**D**) starch content of kenaf seedlings grown in drought conditions. CK (control), D (moderate drought), and D_SA (1 mM SA under moderate drought stress). Values are means ± SD (n = 3). Bars indicate SD. Different letters indicate significant differences in Duncan’s test at *p* ≤ 0.05.

Under drought stress, the content of proline was significantly increased, and the content of proline in group D_SA was higher than in group D. Group D_SA’s proline content, however, is much higher than the CK group’s (Figure 4A). Compared to group CK, the relative electrical conductivity in group D and group D_SA was significantly increased, while the relative electrical conductivity in group D_SA was significantly lower, compared to group D (Figure 4B). Soluble sugar in group D was significantly increased by 32.36% compared with group CK, 15.65% compared with group D_SA, and there was also a significant increase of 14.45% in the D_SA group compared with the CK group (Figure 4C). The soluble starch content, on the other hand, followed the opposite trend as the soluble sugar content (Figure 4D). 

### 3.4. SA Alleviated Drought Stress by Regulating Photosynthesis

**Figure 5:**
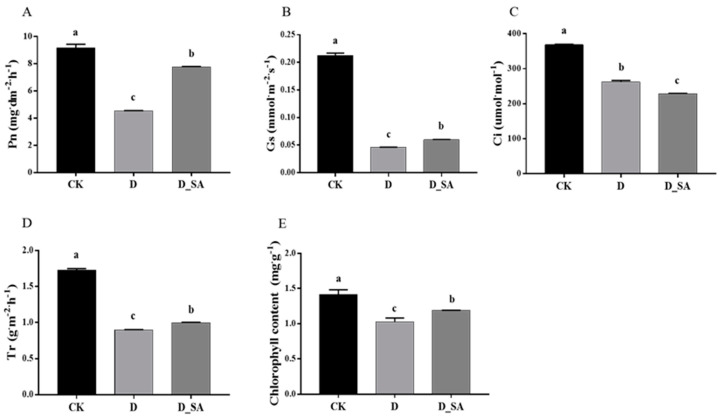
Effects of salicylic acid on (**A**) net photosynthesis (Pn), (**B**) stomatal conductance (Gs), (**C**) intercellular CO_2_ (Ci), (**D**) transpiration rate (Tr), and (**E**) chlorophyll content of kenaf seedlings grown in drought conditions. CK (control), D (moderate drought), and D_SA (1 mM SA under moderate drought stress). Values are means ± SD (n = 3). Bars indicate SD. Different letters indicate significant differences in Duncan’s test at *p* ≤ 0.05.

The net photosynthesis (Pn), stomatal conductance (Gs), intercellular CO_2_ (Ci), and transpiration rate (Tr) of kenaf seedlings in the drought stress group (D) were significantly reduced by 50.21%, 78.37%, 28.79%, and 48.07%, respectively, as compared to the control group. Pn, Gs, Ci, and Tr in the D_SA group were considerably lower than those in the CK group by 15.28%, 71.93%, 38.05%, and 42.28%, respectively. At the same time, the chlorophyll content of the leaves of kenaf seedlings also changed under drought stress. The chlorophyll content of group CK was significantly higher than that of group D and D_SA, and the chlorophyll content of group D_SA was higher than that of group D (Figure 5).

### 3.5. Transcriptome Sequencing, De Novo Assembly, and Functional Annotation

Using the Illumina Hiseq X™ Ten platform, transcriptome analysis of nine samples was completed, and a total of 66.22 Gb of clean data were obtained, with clean data of each sample exceeding 6.45 Gb. The quality assessment of the clean reads revealed that the Q20 and Q30 values were above 97% and 93%, respectively, with an error rate below 0.026%. These metrics suggest high sequencing accuracy. More detailed information regarding the RNA-seq data is presented in Table 1. Overall, the clean reads met the quality standards required for subsequent analyses. Table S1 shows the functional annotations based on the six major databases. All downstream analyses were based on high-quality clean reads. A total of 105,490 transcripts were collected and a total of 71,389 individual genes were further annotated through six publicly accessible databases (NR, Swissport, Pfam, COG, GO, KEGG), yielding 51497 (72.14%), 33400 (46.79%), 14646 (20.52), 38023 (53.26%), 42731 (59.86%), and 18796 (26.33%) genes, respectively.

### 3.6. DEGs in Different Treatment Groups

**Figure 6:**
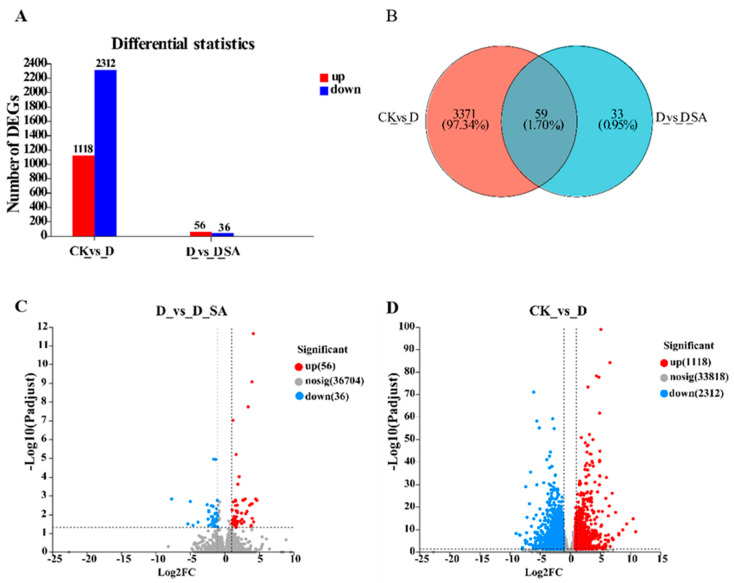
Differentially expressed genes (DEGs) in kenaf seedlings under salicylic acid and drought conditions. (**A**) Statistical chart of differences in expression levels of all genes. (**B**) Volcanic map of CK vs. D expression differences. (**C**) Volcanic map of D vs. D_SA expression differences. (**D**) Venn diagram between two groups. CK (control), D (moderate drought), and D_SA (1 mM SA under moderate drought stress).

In the present study, we used |log2 Fold Change| ≥ 1 and a false discovery rate (FDR)-adjusted *p* value < 0.05 to detect the differentially expressed genes (DEGs) in the three groups. When compared to the control samples, a total of 3430 genes exhibited differential expression in the drought-treated samples (CK vs. D). Among these, 1118 genes (32.59%) were up-regulated, while 2312 genes (67.41%) were down-regulated. At the same time, 92 genes were differentially regulated in the SA treatment group compared with the drought samples (D vs. D_SA). There were 56 (60.87%) up-regulated genes and 36 (39.13%) down-regulated genes (Figure 6A). In addition, 59 genes were highly conserved between the CK vs. D and D vs. D_SA groups, while 3371 genes were found in CK vs. D and 33 genes in D vs. D_SA (Figure 6B). Compared to the control group, the number of down-regulated genes was 2.07 fold higher than that of up-regulated genes, indicating that most of the genes were inhibited under drought stress, while the number of up-regulated genes was 1.56 fold higher than that of down-regulated genes in the SA treatment group, indicating that most of the differential genes were activated under drought stress (Figure 6C,D).

### 3.7. Gene Ontology (GO) Analysis of DEGs

**Figure 7:**
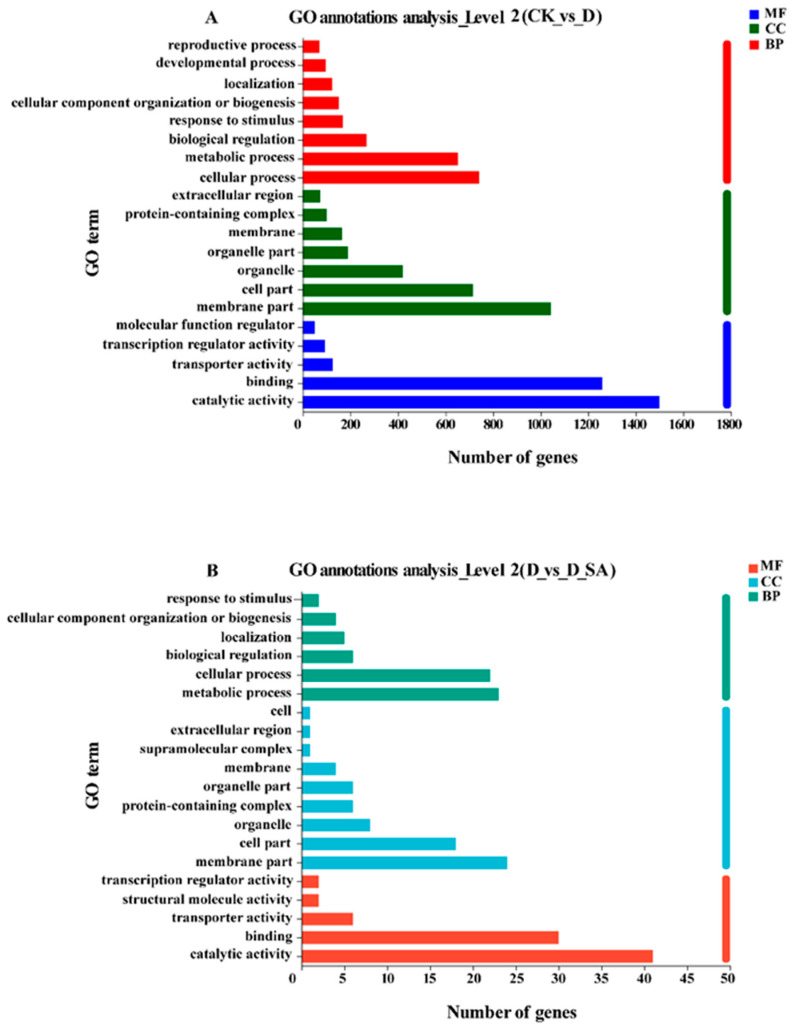
Gene ontology (GO) classification and distribution of GO annotated genes. (**A**) CK vs. D; (**B**) D vs. D_SA. BP, biological process; CC, cellular component; MF, molecular function. CK (control), D (moderate drought), and D_SA (1 mM SA under moderate drought stress).

To elucidate the potential biological functions of DEGs, GO enrichment analysis was performed by GO analysis with a *p* value < 0.05. The results showed that the DEGs of CK vs. D and D vs. D_SA were divided into 47 functional groups, including “biological process” (BP, 22 subcategories), “cellular component” (CC, 12 subcategories), and “molecular function” (MF, 13 subcategories) (Figure 7). In the CK vs. D comparison group, the majority of Gene Ontology (GO) terms were concentrated in the following categories under Biological Process (BP): cellular process, metabolic process, biological regulation, and response to a stimulus. Regarding the Cellular Component (CC) category, the top five subcategories included membrane part, cell part, organelle, organelle part, and membrane. Among the MF category, catalytic activity, binding, and transporter activity are the major subclasses. In group D vs. D_SA, metabolic process, cellular process, and biological regulation were the most important factors in group BP. Within the CC category, membrane part, cell part, and organelle were the predominant components. In the MF category, binding and catalytic activity were the most prominent functions. Significantly, the terms “detoxification”, “stimulus response”, and “signaling” within the BP category, as well as “antioxidant activity” in the MF category, were consistently annotated across both the CK vs. D and D vs. D_SA groups. 1, 170, 3, and 23 DEGs were annotated with the GO terms “detoxification”, “response to stimuli”, “signaling”, and “antioxidant activity”, respectively, and these have been demonstrated to be crucial for plant stress tolerance.

### 3.8. KEGG Pathway Function Analysis of DEGs

**Figure 8:**
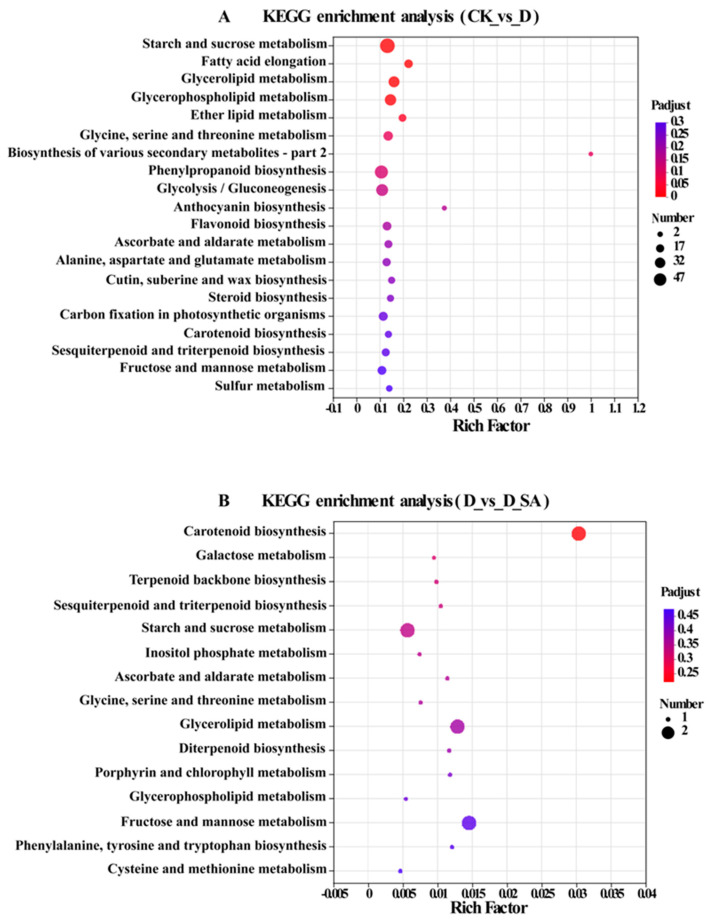
KEGG enrichment of the DEGs. (**A**) CK vs. D; (**B**) D vs. D_SA. CK (control), D (moderate drought), and D_SA (1 mM SA under moderate drought stress).

To characterize the response of biological pathways to drought stress and the mitigating effect of SA treatment on drought stress, all DEGs were assigned to the KEGG functional annotation database. In the group CK vs. D, the KEGG analysis identified that a total of 1070 DEGs were associated with 122 metabolic pathways. These pathways were systematically classified into six primary categories: metabolism, organic systems, environmental information processing, genetic information processing, cellular processes, and human diseases, with subsequent hierarchical division into 19 specialized subclasses (Figure 8). Drought stress causes changes in the subcategories of “carbohydrate metabolism”, “folding, sorting and degradation”, “signal transduction”, “transport and catabolism”, “environmental adaptation”, and “endocrine metabolic diseases”, which represent the largest number of DEGs in these six categories. In addition, DEGs are functionally enriched in the subclasses “carbohydrate metabolic process”, “lipid metabolic process”, and “polysaccharide metabolic process”, which are closely related to the plant response to environmental stimuli and the regulation of growth and development. In the D vs. D_SA group, the KEGG analysis showed that a total of 30 DEGs were associated with 25 pathways. These pathways fall into three categories, namely “metabolism”, “genetic information processing”, and “environmental information processing”. These three categories are further divided into nine subcategories. Under SA treatment, DEGs enriched mainly in “Lipid metabolism”, “Carbohydrate metabolism”, “Metabolism of terpenoids and polyketides”, and “Translation”, which are closely related to plant metabolism and growth and development.

In the above KEGG pathways, “signal transduction”, “carbohydrate metabolism”, “biosynthesis of other secondary metabolites”, “environmental adaptation”, and “folding, sorting and degradation” are the first five ways of KEGG pathways in the CK vs. D group. “Carbohydrate metabolism”, “lipid metabolism”, and “metabolism of terpenoids and polyketides” were the three KEGG pathways in the D vs. D_SA group. “Lipid metabolism” and “metabolism of terpenoids and polyketides” are the KEGG pathways in both groups, including most DEGs (Figure 8).

### 3.9. DEGs Related to Drought Stress Signals in Kenaf

In response to drought stress, a total of 31 DEGs were identified in kenaf, which are involved in the perception and transmission of drought stress signals. Specifically, 15 genes were up-regulated and 16 genes were down-regulated. Among these, 22 DEGs were associated with ABA synthesis and binding, while 9 DEGs functioned as calcium ion receptors. Many DEGs related to ABA signaling pathways exhibited significant up-regulation under drought stress. These genes included 9-cis-epoxide carotenoid dioxygenase (*NCED*), E3 ubiquitin protein ligase *XERICO*, and protein phosphatase 2C (*PP2C*). For most of the above DEGs, there was no difference in gene expression in the D vs. D_SA group, except for the genes, abscisic acid receptor gene *MSTRG.184* (1.67-fold up-regulation) and 9-cis-epoxide carotenoid dioxygenase *(NCED5) MSTRG.28014* (2.27-fold down-regulated) (Table S2).

### 3.10. Transcription Factors (TFs) Participating in Response to Drought Stress in Kenaf

Transcription factors are crucial regulatory proteins that bind to specific nucleotide sequences in the promoter regions of genes, thereby modulating the expression of target genes either by activation or repression. In this study, we identified a total of 67 differentially expressed transcription factors in response to drought stress, with 34 being up-regulated and 33 being down-regulated (Q ≤ 0.05) (Table S3). These genes were *MYB* (9), *TCP* (1), *AP2/ERF* (9), *NF-Y* (4), *Bhlh* (4), *NAC* (2), *AUX/IAA* (6), *C2H2* (1), *C3H* (5), *CAMTA* (3), *G2-like* (4), *HD-ZIP* (4), *B3*(4) and *BSD* (1), and *WRKY* (10) family transcription factors. *NF-Y* gene (*Hca.08G0010830*), *NAC* gene (*MSTRG.23933*), *MYB* gene (*Hca.05G0016340*), 3 *HD-ZIP* genes (*MSTRG.11671*, *MSTRG.4250* and *Hca.04G0029170*), and two *B3* genes (*Hca.06G0040060* and *Hca.08G0017680*) were highly expressed under drought stress. While in the group D vs. D_SA, only two of the above transcription factors were differentially expressed: ethylene-responsive transcription factor 4 (*MSTRG.26004*, 1.39-fold down-regulated), and nucleoid transcription factor Y subunit A-1 (*Hca.08G0010830*, 1.91-fold down-regulated) (Table S3). These results showed that these two transcription factors were sensitive to SA and reduced the damage in kenaf seedlings under drought stress.

### 3.11. DEGs Participate in Drought Stress Response Pathway

In this study, a total of 16 DEGs, including SOD, POD, and GSTs, were identified in association with ROS scavenging. The POD genes (*Hca.02G0008490* and *MSTRG.2228*) and the GST gene (*Hca.03G0040950*) exhibited up-regulation in response to drought stress. However, most DEGs encoding POD were down-regulated under drought stress conditions.

Furthermore, a total of 12 DEGs (3 up-regulated and 9 down-regulated) were identified to be associated with lignin biosynthesis. These DEGs mainly encode 4-coumarate-CoA ligase *(4CL)*, Cinnamoyl-CoA reductase *(CCR),* Caffeoyl-CoA O-methyltransferase *(CCOMT),* and *LACCase*.

In addition, present results demonstrated that other types of DEGs might also be associated with the response of kenaf to drought stress. A total of 12 DEGs were identified (8 up-regulated and 4 down-regulated), which belonged to heat shock proteins (*HSPs*) and late embryonic development abundant proteins (*LEA*). Notably, the DEGs of most late embryogenesis abundant proteins were significantly up-regulated by more than four-fold under drought stress.

Drought stress induces complex changes in the sugar metabolism of plants. In this study, we identified a total of 43 DEGs associated with glucose metabolism, with the majority of these genes being down-regulated. These DEGs are implicated in various metabolic pathways, including starch and sucrose metabolism, fructose metabolism, pentose and glucosidic acid metabolism, galactose metabolism, glycolysis and gluconeogenesis, as well as amino sugar and nucleotide sugar metabolism. In the present study, only two DEGs related to glucose metabolism were found in the above four drought stress response pathway genes in the D vs. D_SA group, which were *Hca.12G0002690* (1.85-fold up-regulated) and *MSTRG.37346* (1.09-fold up-regulated).

The differential genes of the above pathways are shown in the Supplementary Materials (Table S4).

### 3.12. Quantification and Validation of Gene Expression Levels

To confirm the accuracy of the RNA-seq data obtained from kenaf under drought stress, 12 DEGs were randomly selected for validation using qRT-PCR. The qRT-PCR results exhibited a highly consistent trend with the RNA-seq data (Table S5), thereby validating the reliability of the RNA-seq data.

### 3.13. Silencing of HcSABP2 in Kenaf Reduced the Tolerance to Drought Stress

**Figure 9:**
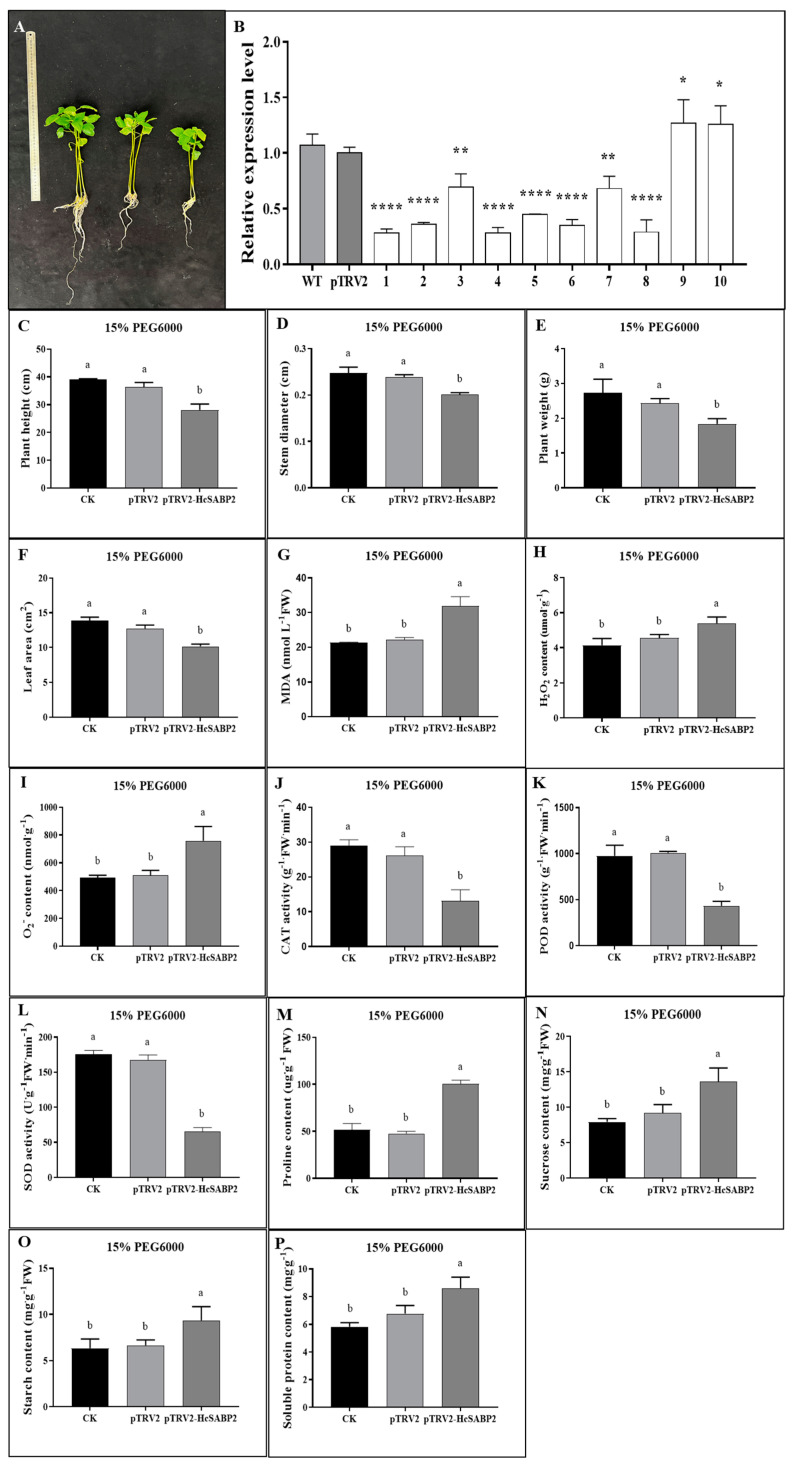
Determination of agronomic traits and physiological traits of kenaf plants silenced with *HcSABP2* under drought stress. (**A**) Morphology difference in VIGS plants under drought treatment; the ruler used in the figure has a maximum measuring range of 50 cm. (**B**) The pTRV2 is the control plant and #1–10 are the *HcSABP2* gene silenced plants. (**C**) Plant height. (**D**) Stem diameter. (**E**) Plant weight. (**F**) Leaf area. (**G**) MDA content. (**H**) H_2_O_2_ content. (**I**) O_2_^−^ content. (**J**) CAT activity. (**K**) POD activity. (**L**) SOD activity. (**M**) Proline content. (**N**) Sucrose content. (**O**) Starch content. (**P**) Soluble protein content. The values are means ± SD (n = 3). Different lowercase letters indicate that a *p* value < 0.05 level difference is significant. * Represents the signifcance level at *p* value < 0.05; ** represents the signifcance level at *p* value < 0.01; **** represents the signifcance level at *p* value < 0.001.

Research on plant SABP2 revealed that it is a highly-affinity salicylic acid binding protein, identified using 3H-salicylic acid as a ligand. SABP2, a soluble protein, belongs to the α/β-hydrolase enzyme family [45]. SABP2 induces defense signaling in ASM-mediated responses [46]. Given the significant changes in the expression of *HcSABP2* under drought stress, it indicates that *HcSABP2* plays a crucial role in drought tolerance in kenaf. In this study, to verify the role of the *HcSABP2* gene in kenaf response to drought stress, VIGS technology was used to silence the gene. The expression level of the *HcSABP2* gene in the silenced plant leaves was detected, and its expression level was significantly lower than that of pTRV2, as shown in Figure 9B. To investigate the role of *HcSABP2* in drought stress, kenaf seedlings silenced with *HcSABP2* were subjected to 15% PEG6000 simulated drought stress for 7 days. As depicted in Figure 9A, following the application of drought stress, various agronomic traits of the plants were assessed. Specifically, no significant differences were observed in plant height (Figure 9C), stem thickness (Figure 9D), fresh weight (Figure 9E), and leaf area (Figure 9F) between the CK and pTRV2 groups. Nevertheless, following the implementation of VIGS to silence *HcSABP2*, there was a notable decrease in all of these characteristics, suggesting that *HcSABP2* might play a crucial role in enhancing drought resistance in kenaf.

To investigate the effect of *HcSABP2* on drought resistance, physiological indicators of *HcSABP2*-silenced plants were measured. Under drought stress, the MDA content, H_2_O_2_ content, and O_2_^−^ content of silenced plants increased by 49.88%, 30.43%, and 54.13%, respectively (Figure 9G–I). The oxidative stress response of kenaf seedlings under drought stress was further studied by analyzing antioxidant enzyme activities. The results showed that under drought stress, the CAT, POD, and SOD activities of silenced plants decreased significantly by 54.59%, 55.64%, and 62.76%, respectively (Figure 9J–L). Meanwhile, under drought stress, the levels of osmotic regulatory substances, such as proline, sucrose, starch, and soluble proteins in the plants also increased significantly, by 94.14%, 71.37%, 47.95%, and 47.68%, respectively (Figure 9M–P). These results indicate that after *HcSABP2* silencing, the antioxidant enzyme activities in kenaf decreased, leading to an increase in ROS levels, which aggravated membrane damage and reduced drought resistance in kenaf.

## 4. Discussion

### 4.1. SA Supports Kenaf Seedlings’ Growth and Photosynthesis with Stronger Antioxidant System Under Drought Stress

Kenaf experienced severe damage from mild drought stress, including wilting, growth retardation, and leaf shedding, that prevented the plant from growing. Although kenaf is a fiber crop and relatively drought-tolerant, severe drought stress still led to a reduction in its production. Plant height, root length, and plant biomass are key indicators for assessing plant tolerance to drought stress. Our results showed that plant growth was significantly inhibited under moderate drought stress. However, a foliar spray of 1 mM SA under drought stress relieved various physiological indexes of kenaf seedlings to a certain extent (Figure 1). These results indicated that an appropriate amount of SA could help kenaf to adapt to moderate drought stress.

The generation and elimination of reactive oxygen species (ROS) are in balance under a normal growth environment. However, drought stress triggered the production of ROS and oxidative damage in plants, leading to reduced plant growth and development. ROS disrupts metabolic processes by damaging DNA and RNA, proteins, lipids, and cells [10].

Under drought stress conditions, several studies have reported an increased activity of antioxidant enzymes, such as SOD, POD, and CAT, which are thought to be related to the accelerated production of reactive oxygen species [47]. The present study showed that SOD, POD, and CAT activities increased under drought stress, indicating that these enzymes should act simultaneously to eliminate H_2_O_2_ and prevent the formation of super-toxic **·**OH. In addition, SOD and POD activities in the D_SA group were lower than those in the D group and higher than those in the CK group, while CAT activities were higher than those in the D group and higher than those in the CK group, indicating that the changes in the above enzyme activities after the application of SA led to a decrease in H_2_O_2_ content compared with the drought treatment group (Figure 2). The results indicated that SA might stimulate the activity of antioxidant enzymes and reduce the accumulation of ROS directly or indirectly. Oxidation induced MDA content, H_2_O_2_ content, and superoxide anion content reflect the ROS status of plants [48]. In the present study, the MDA content, H_2_O_2_ content, and O_2_^−^ content of kenaf seedlings increased under drought treatment, indicating that drought stress triggers the production of reactive oxygen free radicals and alters the structure of the cell membrane, leading to changes in lipid peroxidation within the membrane. Meanwhile, the contents of MDA, H_2_O_2_, and O_2_^−^ in the D_SA group were higher than those in the CK group, but lower than those in the D group. This also reflects that SA can protect kenaf seedlings from drought stress to a certain extent, control the outbreak of ROS, and reduce the damage to kenaf leaves (Figure 3). In this study, SA mitigated the damage caused by drought stress by increasing the proline content and simultaneously decreasing the relative conductivity in kenaf, and relative electrical conductivity indicates the degree of damage to the plant cell membrane [49]. At the same time, SA reduced the soluble sugar content in kenaf under drought stress but increased the soluble starch content (Figure 4). In plants, the concentration of sorbitol in leaves was significantly positively correlated with the activity of Sucrose Phosphate Synthase (*SPS*) and amylase, and negatively correlated with the activity of ADP-glucose-pyrophosphorylase (*ADPGPPase*), while the increase in sorbitol was directly related to the decrease in sucrose and starch [50]. In this study, the expression levels of the *SPS* gene (*Hca.08G0002610*) and amylase genes (*Hca.07G0025810*, *Hca.03G0034380*, and *MSTRG.1062*) were significantly increased. *ADPGPPase* is a key enzyme in starch synthesis. This study did not show the expression level of the *ADPGPPase* gene, so it is speculated that the content of soluble starch may be caused by the decreased activity of *ADPGPPase*. SA-treated maintained ROS homeostasis in vivo. From these physiological parameters, it can be concluded that 1 mM SA foliar spray can make kenaf seedlings have better drought stress tolerance.

According to the above physiological data, SA treatment can alleviate drought stress in kenaf to a certain extent. This is partly reflected by the expression levels of the corresponding genes. Sixty-three genes, including SOD, POD, CAT, APX, GR, and APX, were differentially expressed under drought treatment. The findings indicated that 16 DEGs were significantly up-regulated in the D group. However, 17 DEGs were significantly up-regulated in the D_SA group (Table S6). It is interesting to note that compared to the CK group, the expression levels of most genes in both the D and D_SA groups were increased, but the differential genes in the D group were more significant, indicating that when kenaf seedlings were treated with drought, the activity of antioxidant enzymes decreased and the content of non-enzymatic substances increased except for CAT.

Photosynthesis plays an important role in the cycling of material and the flow of energy throughout the ecosystem. Water is a raw material for photosynthesis, and drought naturally inhibits photosynthesis [51]. A previous study indicated that as drought stress increases, chlorophyll synthesis in plants is impaired and chlorophyll content decreases [52]. The higher the level of drought stress, the lower the photosynthetic rate of crops tends to be. Water stress reduces stomatal conductance (Gs) and intercellular CO_2_ concentration (Ci), which impairs photosynthetic efficiency [53]. It has been found that water stress can lead to a non-reversible decrease in net photosynthetic rate (Pn), transpiration rate (Tr), and prolongation of the inefficient Pn period in maize [54]. The results of this experiment showed that the Pn, Gs, Ci, and Tr of photosynthesis under drought stress showed similar results as previous studies (Figure 5), and the photosynthetic capacity of kenaf leaves could be improved to a certain extent when the leaf surface was sprayed with SA.

Photosynthesis in plants is more sensitive to stress. The PSBQ gene, encoding the one core protein in the reaction center of PSII and PSI, plays an important role in the process of chlorophyll degradation [55]. *HIPP* plays a role in enhancing photosynthetic pigments in plant photosynthesis [56]. In this study, one *NAC* gene (*Hca.13G0005380*) and three *HIPP* genes (*Hca.01G0011720*, *Hca.17G0014990,* and *Hca.12G0000520*) and one *PSBQ* (*MSTRG.7141*) were up-regulated under drought treatment compared with the control group. Compared with the CK group, there was no significant difference in the expression level of *PSBQ* (*MSTRG.7141*) between the D group and the D_SA group. Since *PSBQ* is involved in chlorophyll degradation, it is speculated that SA can slow down the degradation of chlorophyll in kenaf leaves, thus maintaining photosynthesis and protecting plants from the effects of drought stress. Correspondingly, in group D, the contents of Pn, Gs, Ci, Tr, and chlorophyll in kenaf leaves were lower than those in group CK, while in group D_SA, except Ci, the contents of Pn, Gs, Tr, and chlorophyll in kenaf leaves were higher than those in group D.

### 4.2. Defense Response of Kenaf to Drought Stress

Lignin, a key component of the plant cell wall, can have its content and composition altered by drought stress. Lignin deposition is a common response of plants to abiotic stress [57]. Cinnamyl-CoA reductase (*CCR*) is a key enzyme in the lignin biosynthesis pathway, which features a complex, grid-like structure and involves multiple substrates [58]. Research has demonstrated that the expression of the *CCR* gene is up-regulated in response to drought stress [59], and Caffeoyl Shikimate Esterase (*CSE*) activity hydrolyzes the product cafeylshikimate back to the corresponding carboxylic acid level. In this study, two *CCR* genes (*Hca.18G0005460* and *MSTRG.15294*) and one *CSE* gene (*Hca.15G0021770*) were up-regulated in group D. In the D_SA group, the expression of the above genes was also up-regulated, but there was no significant difference compared with the D group. However, the impact of drought stress on lignin biosynthesis remains poorly understood.

Under drought stress, significant changes in sugar distribution, metabolism, and transport occurred in plants [60]. It has been documented that non-structural carbohydrates, including sucrose and sugar alcohols, tend to accumulate within cells, thereby enhancing osmotic stress tolerance during drought conditions [61]. Zanella et al. [62] noted that β-amylase promotes the conversion of starch into soluble sugars and described the overexpression of *AtADH1* in *Arabidopsis*. In Arabidopsis, the accumulation of total soluble sugars is enhanced, which in turn, boosts its resistance to abiotic stress [63]. This study raised two beta-amylase (*MSTRG.1062* and *Hca.14G0013700*) and one *ADH1* (*Hca.13G0008030*), which may indicate that kenaf seedlings can adapt to drought stress by increasing soluble sugar. These results suggest that SA may alleviate the effects of drought stress by changing the glucose metabolism process. In addition, DEGs related to raffinose metabolism also changed significantly in kenaf seedlings under drought stress. In general, kenaf seedlings can enhance cell wall stability and increase total cellular soluble sugars in response to short-term water scarcity. Only two genes (*Hca.12G0002690* and *MSTRG.37346*) in the above DEGs showed differences under the conditions of drought and SA treatment, indicating that the changes in kenaf plant sugar under the conditions of SA treatment may be related to these two genes compared to the drought treatment group.

Under drought stress conditions, the expression of numerous genes encoding drought stress-related proteins was significantly up-regulated in kenaf, including those for *LEA* and *HSP* (Table S5). LEA proteins, which are highly hydrophilic, play a crucial role in enhancing plant tolerance to water deficit. In *Arabidopsis*, plants overexpressing *LEA2* show higher drought tolerance and oxidative stress than wild-type plants [64]. Likewise, the overexpression of *OSLEA3-2* has been shown to improve the drought tolerance of rice under water stress conditions [65]. Similarly, the overexpression of *OSLEA3-2* enhanced the drought tolerance of rice to water stress [65]. As molecular chaperones, *HSPs* play a role in enabling living plants to maintain cellular stability under environmental stresses, such as drought, high temperature, and salinity [66]. Prior research has indicated that small heat shock proteins (*Hsp20*) are responsive to drought stress. For instance, the CaHsp25.9 protein has been shown to actively regulate drought tolerance in pepper plants [67], and the overexpression of *ZmHSP16.9* enhances drought tolerance in tobacco [68]. Thus, functional proteins are crucial in the mechanisms underlying plant drought resistance. In the drought plus SA treatment group, compared with the control group, the above trends of DEGs in response to protein stress were consistent with those in the drought group, indicating that the protein genes in response to drought stress played their normal functions under SA treatment. The heat map of the genes involved in the defense pathway of kenaf against drought stress is shown in Figure S2, and the expression levels of most genes in the D_SA group were between the CK group and the D group.

### 4.3. Plant Signaling Pathway Plays an Important Role in Kenaf Response to Drought Stress

Regulation of drought tolerance in plants is a complex process involving many genes. Under drought stress, the plant cell membrane perceives stress signals, which subsequently trigger and activate secondary messengers, such as Ca²⁺ and ABA, to modulate the expression of downstream genes. The ABA signaling pathway is an important signal transduction pathway in the drought stress signaling pathway. *NCED* serves as a crucial rate-limiting enzyme in the ABA biosynthesis pathway. It catalyzes the oxidative cleavage of the epoxide carotenoid 9-cis isomer, converting 9-cis-meso-zeaxanthin and 9′-cis-neoxanthin into xanthoxin. This enzyme is frequently up-regulated under stress conditions to enhance ABA synthesis [69]. When intracellular ABA levels increase, the ABA receptor PYR/PYL/RCAR protein binds to *PP2C*, thereby releasing *SnRK2;* subsequently, *SnRK2* phosphorylates downstream substrates and actively activates the ABA response [70]. *SnRK2s* is a major protein kinase that transmits ABA signals throughout the plant. There were two *NCED* genes (*MSTRG.28014* and *Hca.13G0005170*), one SnRK gene (*MSTRG.4158.1*), and six *PP2C* genes in the D vs. CK transcriptome (*Hca.18G0001370*, *MSTRG.37258*, *MSTRg.1668*, *Hca.02G0020130*, *Hca.02G0001720,* and *Hca.01G0029750*) that were significantly up-regulated under drought stress. At the same time, only the Abscisic acid receptor *PYL5* (*MSTRG.184*) and *NCED5* (*MSTRG.28014*) showed significant differences in ABA signal transduction pathway genes between drought and SA-treated DEGs. Based on these observations, we suggest that ABA signaling plays a significant role in the transduction of drought stress signals. It is well established that SA and ABA synergistically contribute to plant responses to drought stress. In this study, we concurrently quantified the levels of SA under drought conditions and observed a significant increase in SA content relative to the control group (CK). This increase is congruent with the up-regulation of ABA synthesis-related genes, suggesting that both ABA and SA act in concert within kenaf plants to mitigate the detrimental effects of drought stress (Figure S3).

JA plays a significant role in plant stress responses, particularly under abiotic stress conditions, such as drought. As indicated in the literature, JA can enhance plant stress tolerance through the JA signaling pathway under various adverse environmental conditions [71]. Generally, the signaling pathways of JA and SA exhibit antagonistic interactions, and this interplay helps induce plant resistance to a range of stresses [72]. Prior research has explored the mechanisms underlying the cross-talk between the JA and SA signaling pathways, revealing that several genes are involved in mediating the antagonistic effects between SA and JA, including MYC2, *PDF 1.2* (Plant Defensin 1.2), *TGAs* (TF family) [73], *MAPK* (Mitogen-Activated Protein Kinase), *NPR1*, *ERF1*, *WRKY62*, *WRKY70*, *GRX480* (Glutaredoxin 480), *ORA59* (Oleate-Responsive AP2/ERF 59), and *JAZ* [74]. The existence of the ortholog *NPR1* in the common ancestor of all land plants implies that the cross-talk between the JA and SA signaling pathways may be a universal phenomenon in plants [75]. In this study, compared to the control group (CK), the treatment of the other two groups led to an increase in SA content, while in the drought group, the expression of JA biosynthesis-related genes LOX14 and OPR3 was significantly reduced, and the expression of MYC2 was significantly up-regulated. This suggests that SA-JA cross-talk occurs in the process of kenaf resisting drought stress, and the relationship is antagonistic.

### 4.4. Response of Transcription Factors to Drought Stress

Transcription factors exert a significant influence on drought stress responses through the regulation of specific downstream genes. The overexpression of *PebHLH35* in Arabidopsis exhibited enhanced tolerance to water stress [76], and when *MbMYB108* was introduced into *Arabidopsis*, it greatly improved the cold and drought tolerance of transgenic plants [77]. Persak and Pitzschke [78] discovered that the overexpression of *MYB44-REP* in *Arabidopsis* led to reduced tolerance to drought stress. Additionally, the *AtMYB2* protein has been reported to participate in ABA-induced gene expression under drought stress conditions [79]. The gene encoding *DREB2A* exhibited strong induction in response to drought stress, and its overexpression was found to significantly enhance drought tolerance [80]. In addition, genes from the *NAC*, *bZIP*, and *bHLH* families have also been reported to be involved in many drought stress responses [81]. Based on the expression of transcription factors in different families under drought induction, it can be inferred that transcription factors play a central role in the complex drought response regulatory network of kenaf. At the same time, compared with the drought treatment group, the gene expressions of the above transcription factors, only two DEGs, *AP2/ERF* (*MSTRG.26004*) and *NF-Y* (*Hca.08G0010830*), were significantly decreased in the SA treatment group under drought stress, indicating the key roles in the process of alleviating drought stress with SA.

### 4.5. Effect of Exogenous SA on Stress Resistance in Kenaf

**Figure 10:**
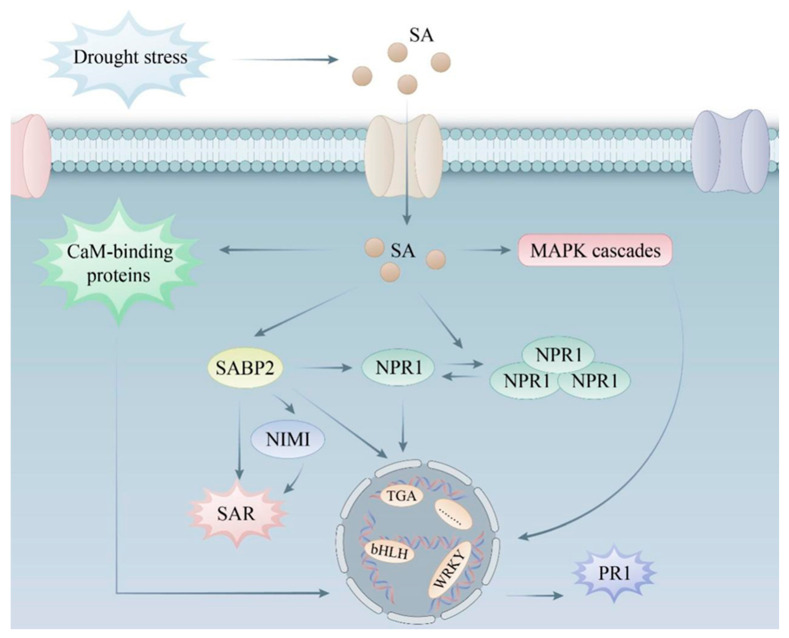
Signaling pathway of SA resistance to drought stress.

Farzana Latif et al. [82] showed that the mechanism underlying SA-induced drought tolerance in maize involves the accumulation of soluble and cell wall-bound phenolic compounds through the foliar application of SA. Lamb et al. [83] showed that SA can rapidly induce the accumulation of ROS in plant tissues, activate plant SAR, and play an important role in resisting external stress. The findings of this study also indicated that the application of SA under drought stress conditions can help mitigate stress and support the normal growth of plants. Non-inducible gene (*NPR1/NIMI*) is an important positive regulator of SAR response induced by SA. Cao et al. [84] found that Arabidopsis mutant (*npr1*) could not produce the SAR response, and the expression of resistance genes was minimal, but the SA accumulated in vivo was comparable to that of the wild-type. These results indicate that the NPR1 gene is an indispensable regulator of SAR production in plants, and it is upstream of the expression of resistance genes. When plants are under external stress, *Enhanced Disease Susceptibility 1 (EDS1)* and *Phytoalexin Deficiency 4 (PAD4)* will be stimulated to promote the synthesis of SA [85], and then SA binds to salicylate binding protein (SABP), forming a complex that transmits information to intracellular signaling molecules, regulates the intracellular balance between oxidation and reduction levels, and enables aggregated *NPR1* in the cytoplasm to form its monomeric form. Monomeric NPR1 is transported into the nucleus through intracellular transport. It acts on *TGAs*, *SNI1*, *WRKYs,* and other transcription factors, and finally, induces the expression of defense genes, such as PR, and cells acquire disease resistance [86]. In addition, there is an *NPR1*-independent SA signaling pathway in plant cells, in which Ca^2+^-CaM, MAPK kinase, and other signaling molecules play important roles [87]. SA can induce a series of disease course related proteins (PRs), and *PR1* expression in tobacco and *Arabidopsis* is only induced by SA [88], so *PR1* is considered a marker protein of SA-induced SAR. The signal transduction pathway of SA in cells is shown in Figure 10.

In the present study, the increased expression of the *SABP2* gene increased due to foliar spray of SA treatment under drought stress, indicating that SA binds to *SABP2* to transmit the information and activate SAR when plants are under stress conditions. However, under drought stress, the expression of *SABP2* did not increase or even decrease. It is speculated that the signal pathway of the SA relief pathway in plants was seriously damaged due to the high-stress level, failing in information transmission. In the present study, the expression of *NPR1* was decreased in both drought treatment and drought treatment with SA spraying, and the down-regulation was greater in drought treatment with SA spraying. It is speculated that the SAR response induced by *NPR1* is not solely dependent on the transient expression of the *NPR1* gene. It is more likely that the aggregated NPR1 forms its monomer *NPR1* due to *SABP2*, thus realizing the rapid accumulation of NPR1. Under drought conditions, the expression levels of *TGA4*, *WRKY28*, *WRKY65,* and *bHLH137* increased, which were 2.65, 9.65, 3.62, and 7.59 times, respectively. However, in the drought group treated with SA, the above transcription factors increased to 1.56, 6.31, 2.74, and 5.14 times, and the gene expression levels were all lower than those in the drought group. The heat map of the above gene expression is shown in Figure S4. It is speculated that exogenous SA was applied to reduce the synthesis of endogenous SA in plants. To some extent, the expression of transcription factors in the signaling pathway was up-regulated less than that in the drought-treated group without SA. Then, the expression of PR and other defense genes is induced, and the cells acquire disease resistance. Finally, SA can induce a series of disease course related proteins (PRs) to alleviate the damage of drought stress on plants.

## 5. Conclusions

Drought stress affects the growth and development of kenaf, and SA could effectively relieve the pressure on kenaf seedlings under drought stress in terms of improved agronomic traits, antioxidant systems, osmotic substances, photosynthesis, reduced H_2_O_2_, relative electrical conductivity, and MDA content. At the same time, transcriptome analysis showed that many DEGs and their involved pathways, such as those of antioxidant systems, signaling pathways, transcription factors, and defense response to drought, were related to the drought stress response. Our findings provided new insights into the molecular mechanism of salicylic acid-alleviated drought stress in kenaf.

## Figures and Tables

**Table 1:** Summary of RNA-seq data.

Sample	Raw Reads	Raw Bases	Clean Reads	Clean Bases	Error Rate (%)	Q20 (%)	Q30 (%)	GC Content (%)
CK_1	55107504	8321233104	54677748	7984559342	0.0253	97.9	93.9	46.98
CK_2	46824708	7070530908	46509826	6815871754	0.0249	98.05	94.2	46.94
CK_3	49450228	7466984428	49137406	7222389650	0.0251	97.99	94.07	46.98
D_1	53846512	8130823312	53501434	7792191587	0.0247	98.13	94.42	46.69
D_2	57894746	8742106646	57527896	8367660141	0.0248	98.13	94.37	46.86
D_3	44973020	6790926020	44591542	6589047419	0.0249	98.06	94.25	46.32
D_SA_1	43838926	6619677826	43551168	6447894248	0.0248	98.10	94.34	46.72
D_SA_2	50601642	7640847942	50262802	7353139934	0.0248	98.09	94.31	46.47
D_SA_3	52796812	7972318612	52465498	7650982656	0.025	98.04	94.18	46.72

## Data Availability

The original contributions presented in the study are included in the article/Supplementary Material, further inquiries can be directed to the corresponding author.

## Supplementary Materials

The following supporting information can be downloaded at: https://www.mdpi.com/article/10.3390/life15020281/s1, Figure S1: Growth morphology and biomass of kenaf seedlings sprayed with different concentrations of salicylic acid under moderate drought conditions; Figure S2: Heat map of genes associated with defense response to drought stress; Figure S3: Determination of salicylic acid content; Figure S4: Heat map of salicylic acid-related genes; Figure S5: F-values of ANOVA for all data; Table S1: Transcriptome functional annotation; Table S2: DEGs related to drought signal; Table S3: Transcription factors response to drought stress; Table S4: DEGs involved in defense responses to drought stress; Table S5: Confirmation of the expression profiles of selected genes by qRT-PCR; Table S6: Expression levels of ROS related genes; Table S7: Primers of qRT-PCR and VIGS.
