# Supplementary material for: Physiological and Transcriptome Analysis Reveal the Underlying Mechanism of Salicylic Acid-Alleviated Drought Stress in Kenaf (Hibiscus cannabinus L.)
Source: Life (Basel). 2025 Feb 12;15(2):281. doi: 10.3390/life15020281 (PMC11856667; doi:10.3390/life15020281)
Supplement: Supplementary file 1 [file life-15-00281-s001.zip › Fig. S3.docx]

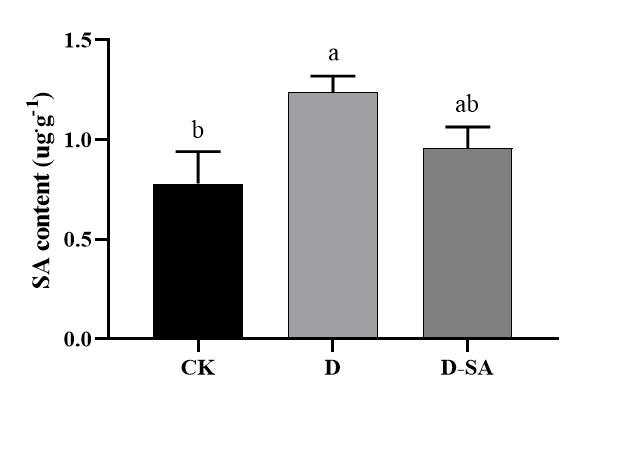


Fig. S3. Determination of salicylic acid content. Values are means ± SD (n = 3). Bars indicate SD. Different letters indicate significant differences of Duncan’s test at *P* ≤ 0.05.
