# Supplementary material for: Physiological and Transcriptome Analysis Reveal the Underlying Mechanism of Salicylic Acid-Alleviated Drought Stress in Kenaf (Hibiscus cannabinus L.)
Source: Life (Basel). 2025 Feb 12;15(2):281. doi: 10.3390/life15020281 (PMC11856667; doi:10.3390/life15020281)
Supplement: Supplementary file 1 [file life-15-00281-s001.zip › Fig. S5.docx]

**F-values of ANOVA for all data**


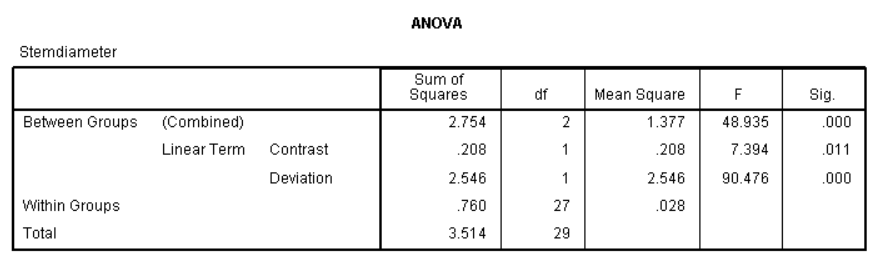


Fig. 1-(B): Stem diameter


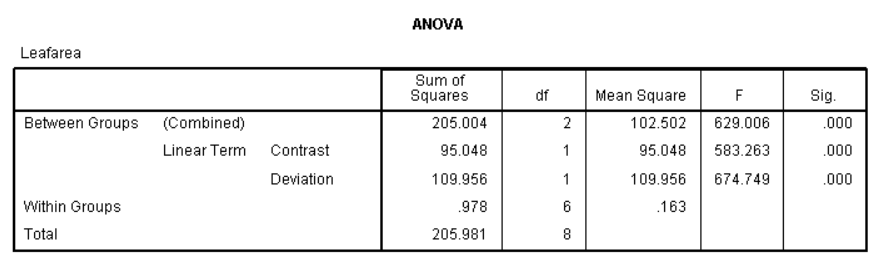


Fig. 1-(C) Leaf area


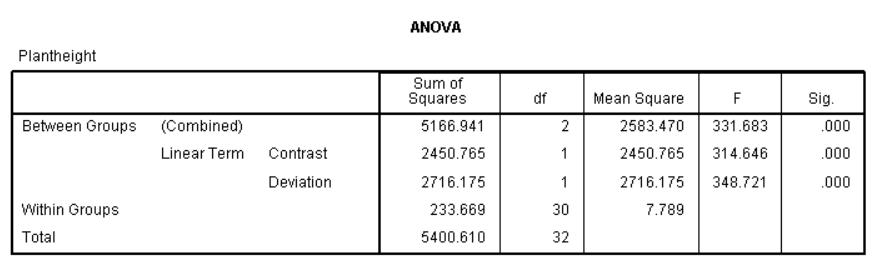


Fig. 1-(E) Plant height


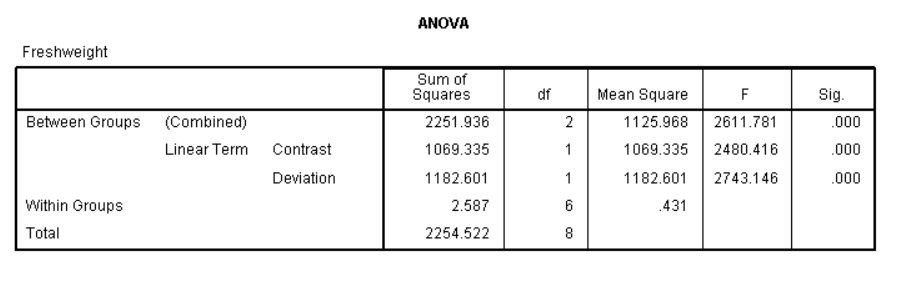


Fig. 1-(F) Fresh weight


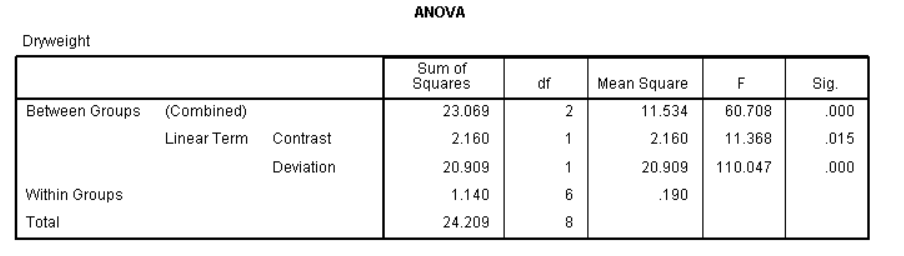


Fig. 1-(G) Dry weight


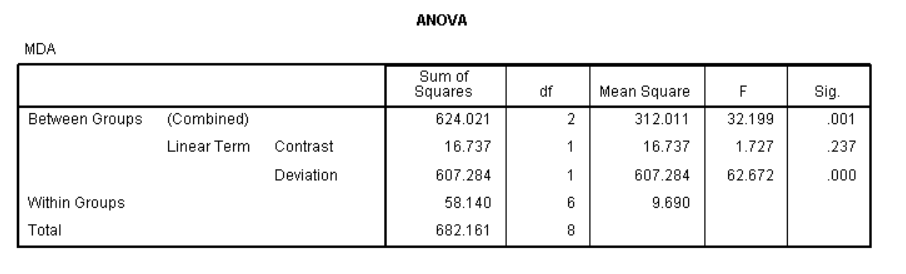


Fig. 2-(A) malondialdehyde (MDA) content


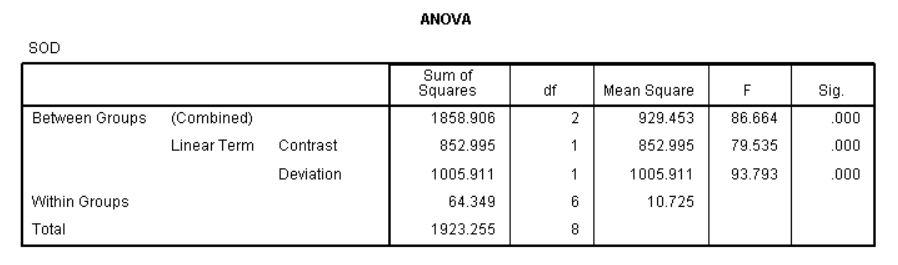


Fig. 2-(B) superoxide dismutase (SOD) activity


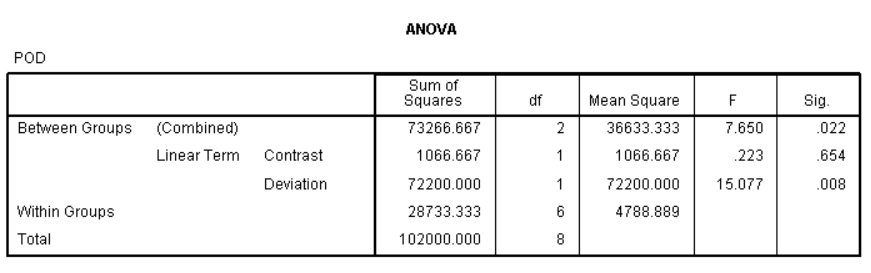


Fig. 2-(C) peroxidase (POD) activity


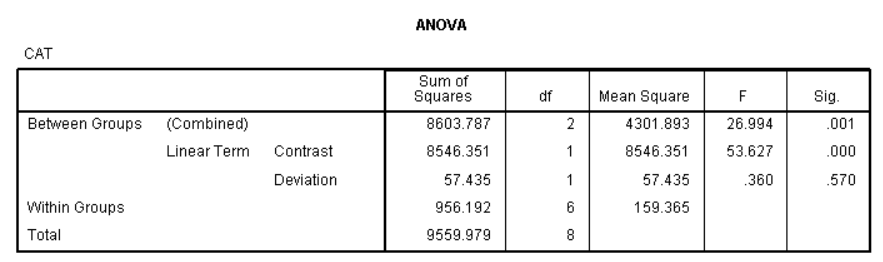


Fig. 2-(D) catalase (CAT) activity


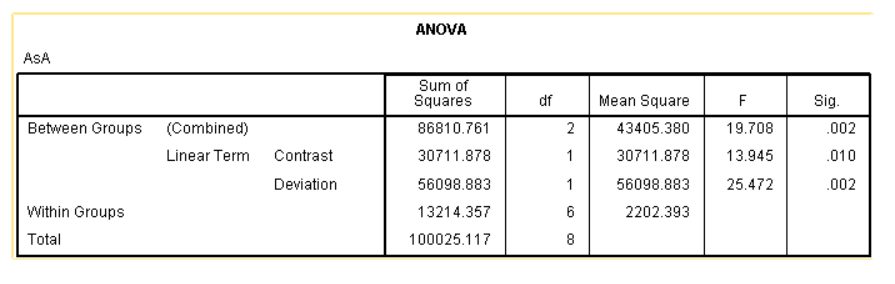


Fig. 2-(E) AsA content


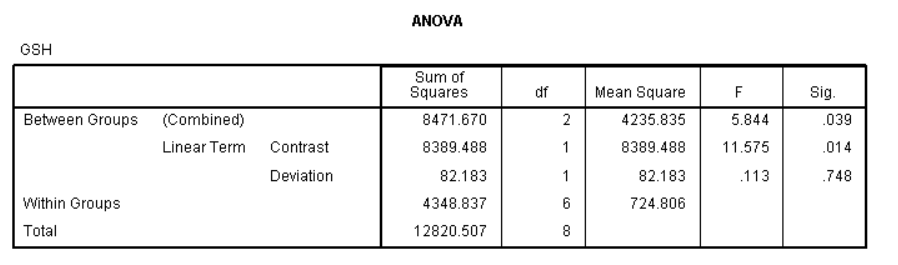


Fig. 2-(F) glutathione (GSH) content


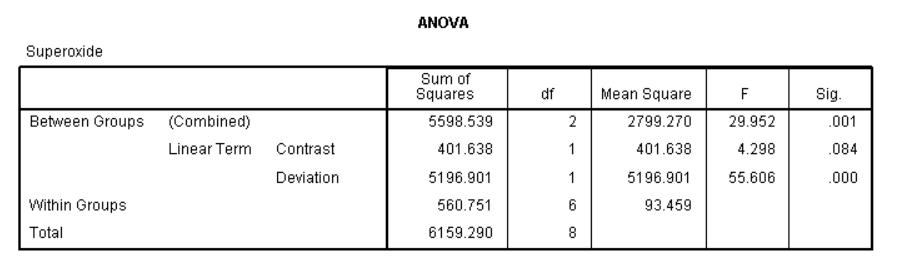


Fig. 2-(G) Superoxide action content


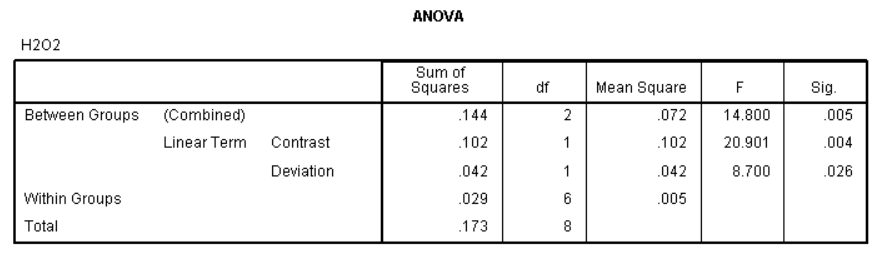


Fig. 2-(H) H_2_O_2_ content


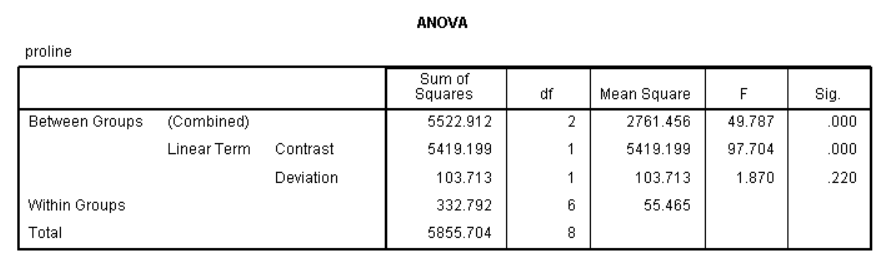


Fig. 4-(A) Proline content


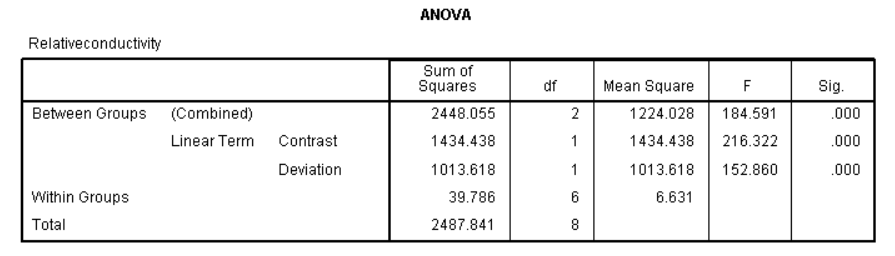


Fig. 4-(B) Relative conductivity


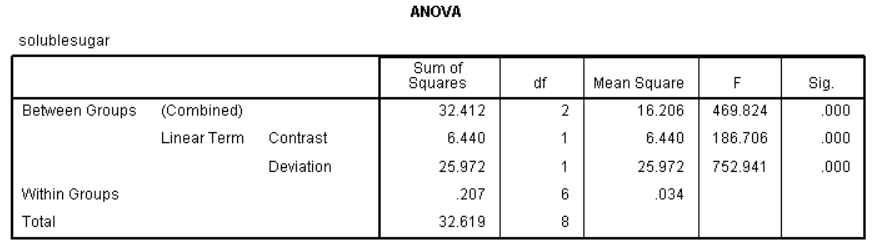


Fig. 4-(C) Soluble sugar content


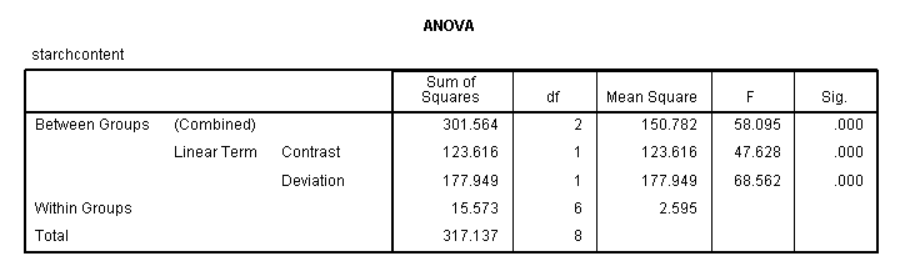


Fig. 4-(D) Starch content


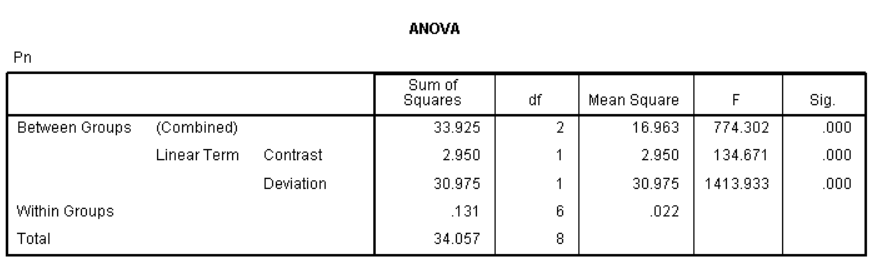


Fig. 5-(A) net photosynthesis (Pn)


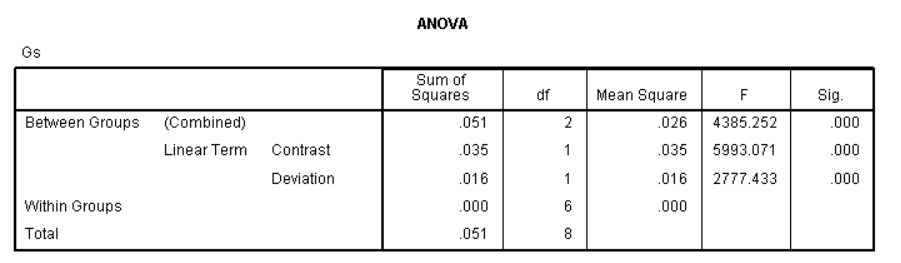


Fig. 5-(B) stomatal conductance (Gs)


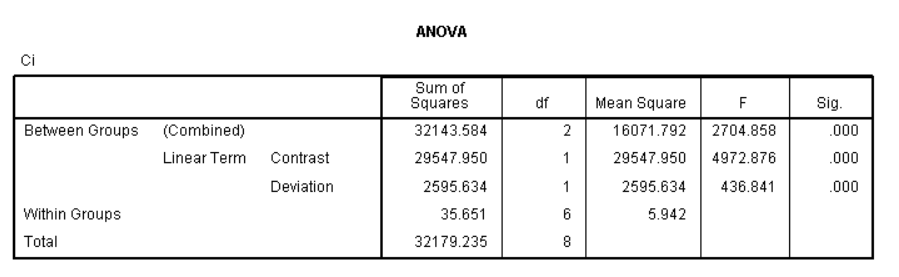


Fig. 5-(C) intercellular CO_2_ (Ci)


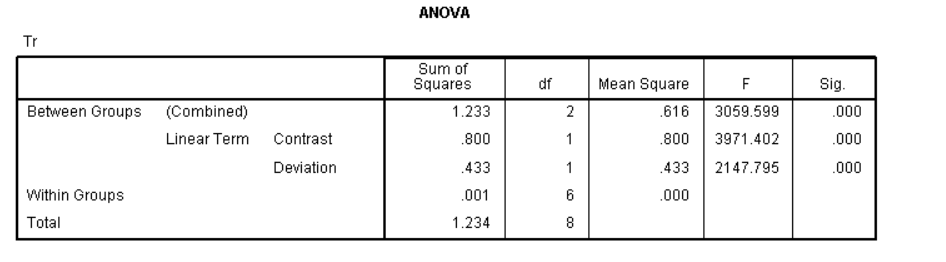


Fig. 5-(D) transpiration rate (Tr)


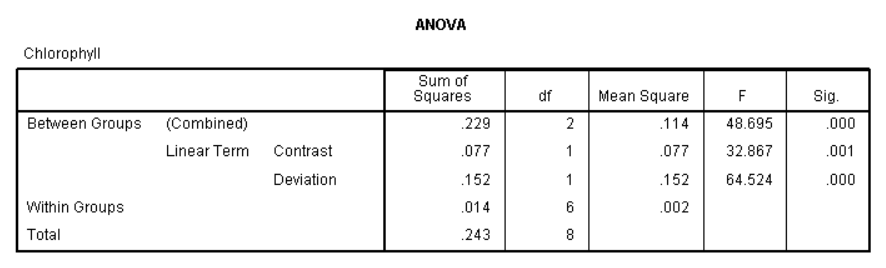


Fig. 5-(E) total chlorophyll content


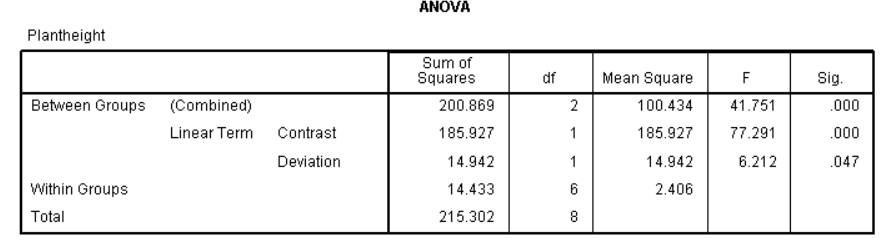


Fig. 9-(C) Plant height


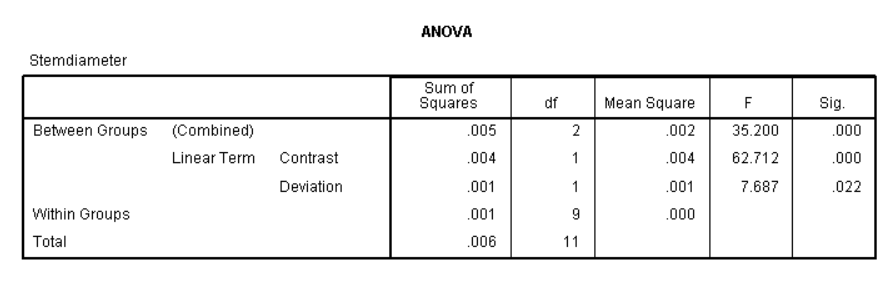


Fig. 9-(D) Stem diameter


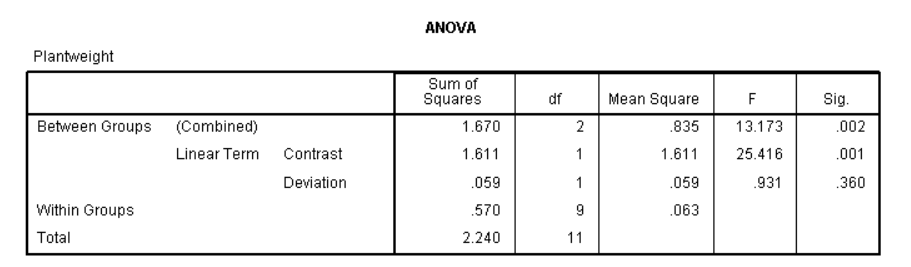


Fig. 9-(E) Plant weight


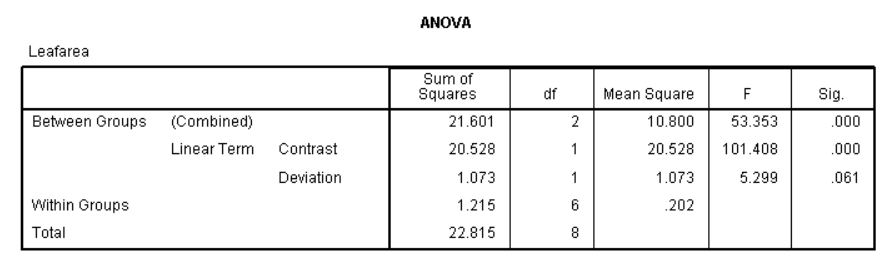


Fig. 9-(F) Leaf area


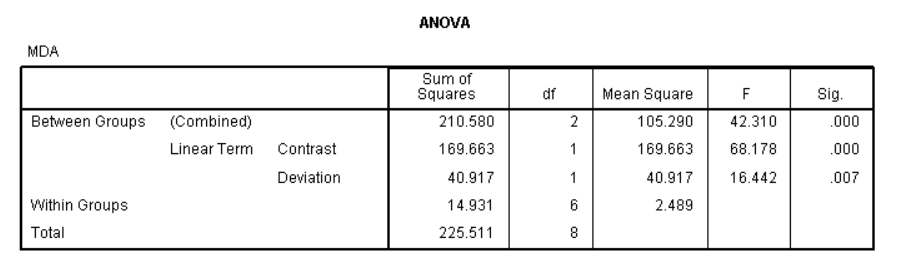


Fig. 9-(G) MDA content


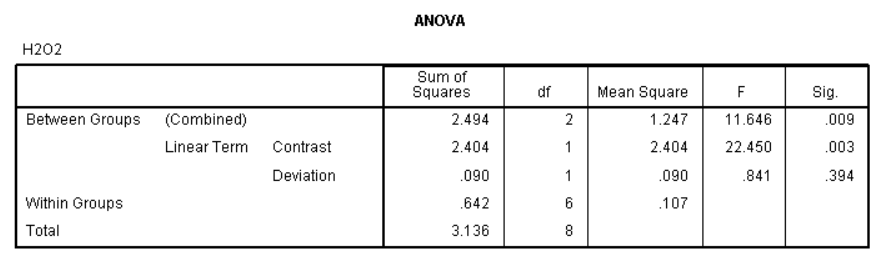


Fig. 9-(H) H_2_O_2_ content


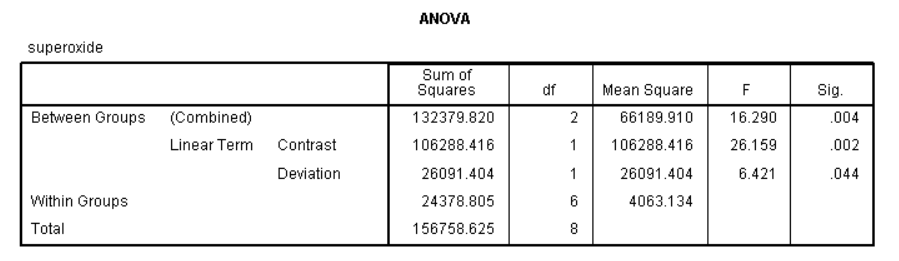


Fig. 9-(I) O_2_^−^ content


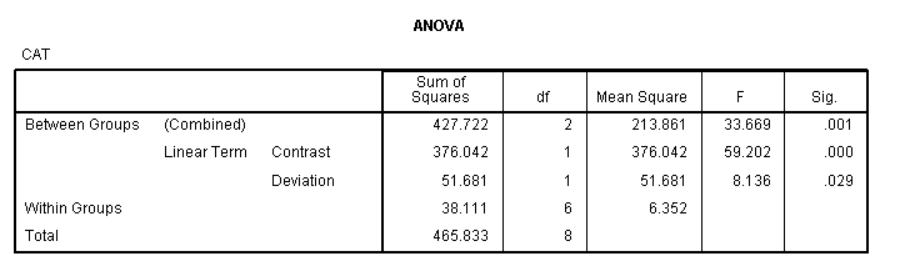


Fig. 9-(J) CAT activity


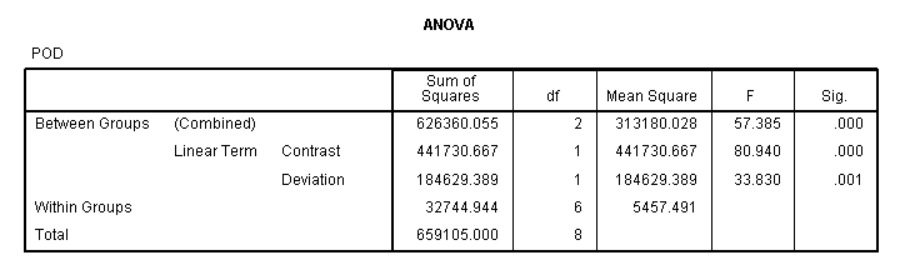


Fig. 9-(K) POD activity


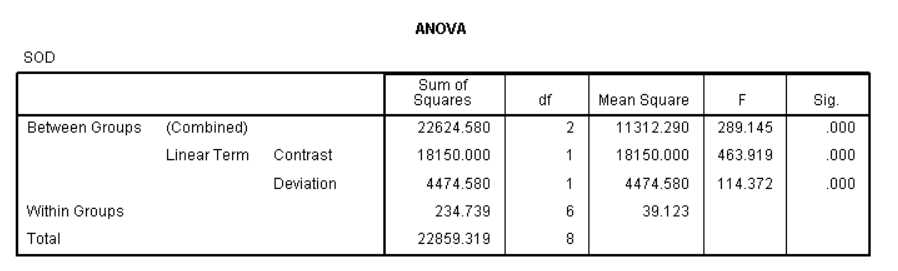


Fig. 9-(L) SOD activity


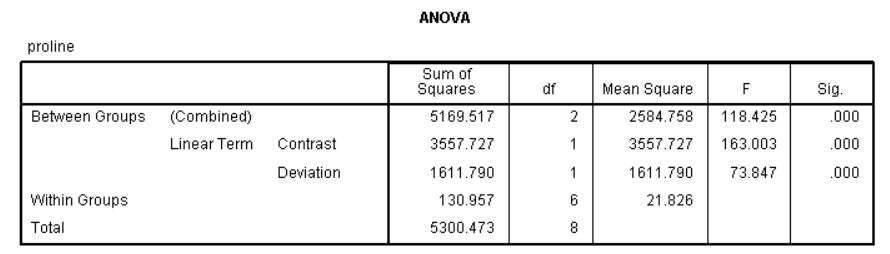


Fig. 9-(M) Proline content


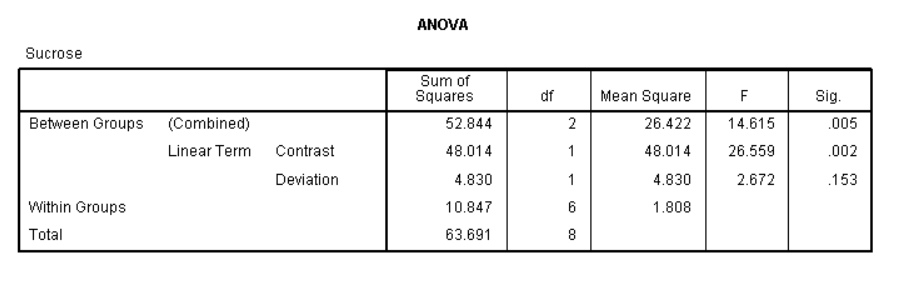


Fig. 9-(N) Sucrose content


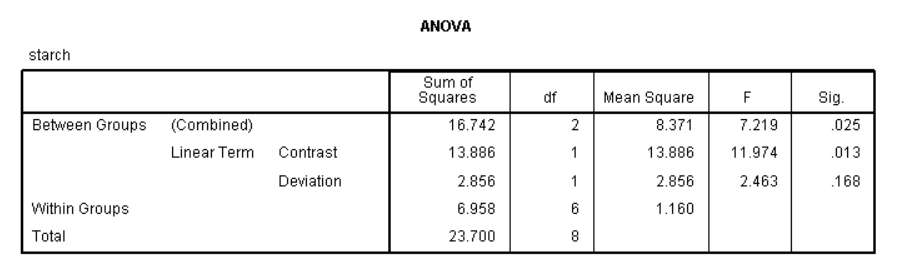


Fig. 9-(O) Starch content


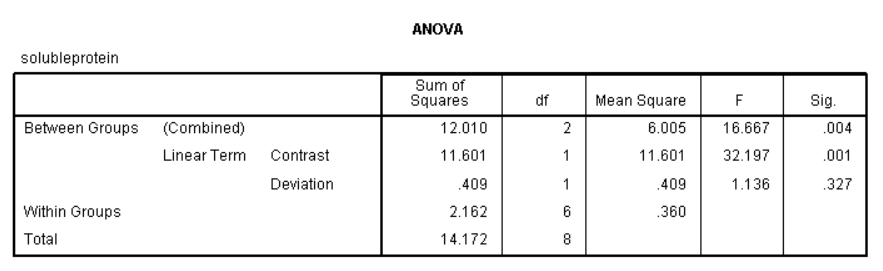


Fig. 9-(P) Soluble protein content
