# Supplementary material for: Physiological and Transcriptome Analysis Reveal the Underlying Mechanism of Salicylic Acid-Alleviated Drought Stress in Kenaf (Hibiscus cannabinus L.)
Source: Life (Basel). 2025 Feb 12;15(2):281. doi: 10.3390/life15020281 (PMC11856667; doi:10.3390/life15020281)
Supplement: Supplementary file 1 [file life-15-00281-s001.zip › Table S1.docx]

**Table S1** Transcriptome functional annotation

|  | Expre_Gene number（percent） | Expre_Transcript number（percent） | All_Gene number（percent） | All_Transcript number（percent） |
| --- | --- | --- | --- | --- |
| GO | 27376 (0.7124) | 25454 (0.7296) | 38807 (0.5903) | 38807 (0.5903) |
| KEGG | 13859 (0.3607) | 12820 (0.3674) | 16828 (0.256) | 16828 (0.256) |
| COG | 26169 (0.681) | 24353 (0.698) | 33853 (0.515) | 33853 (0.515) |
| NR | 32537 (0.8467) | 30228 (0.8664) | 46679 (0.7101) | 46679 (0.7101) |
| Swiss-Prot | 23346 (0.6075) | 21780 (0.6242) | 29679 (0.4515) | 29679 (0.4515) |
| Pfam | 7778 (0.2024) | 7342 (0.2104) | 10893 (0.1657) | 10893 (0.1657) |
| Total_anno | 32625 (0.849) | 30300 (0.8684) | 46889 (0.7132) | 46889 (0.7132) |
| Total | 38427 (1.0) | 34890 (1.0) | 65740 (1.0) | 65740 (1.0) |
