# Supplementary material for: Physiological and Transcriptome Analysis Reveal the Underlying Mechanism of Salicylic Acid-Alleviated Drought Stress in Kenaf (Hibiscus cannabinus L.)
Source: Life (Basel). 2025 Feb 12;15(2):281. doi: 10.3390/life15020281 (PMC11856667; doi:10.3390/life15020281)
Supplement: Supplementary file 1 [file life-15-00281-s001.zip › Table S2.docx]

**Suppl. Table S2** DEGs related to drought signal in CKvsD

| Gene ID | Log2(Fold Change) | Up/Down | Description |
| --- | --- | --- | --- |
| MSTRG.184 | -1.78548 | down | Abscisic acid receptor PYL5 |
| Hca.12G0008280 | -5.07735 | down | abscisic acid receptor PYL4-like |
| MSTRG.12109 | -1.42886 | down | Abscisic acid receptor PYL1 |
| Hca.12G0019900 | -1.05074 | down | abscisic acid receptor PYL2-like |
| MSTRG.37054 | 4.477721 | up | Probable E3 ubiquitin-protein ligase XERICO |
| Hca.03G0045660 | -2.86541 | down | probable protein phosphatase 2C 22 isoform X2 |
| Hca.18G0001370 | 1.45326 | up | protein phosphatase 2C 77 |
| MSTRG.37258 | 2.112968 | up | putative protein phosphatase 2C 39 |
| Hca.06G0021540 | -4.45775 | down | phosphatase 2C (PP2C)-like protein |
| MSTRG.1668 | 2.53481 | up | putative protein phosphatase 2C 75 |
| Hca.03G0007690 | -1.39423 | down | probable protein phosphatase 2C 15 |
| Hca.05G0001470 | -1.37593 | down | probable protein phosphatase 2C 49 |
| MSTRG.33255 | -1.43234 | down | putative protein phosphatase 2C 15 |
| Hca.02G0020130 | 1.067754 | up | probable protein phosphatase 2C 25 isoform X1 |
| Hca.02G0001720 | 1.120619 | up | Phosphatase 2C 49 |
| Hca.01G0029750 | 1.072828 | up | probable protein phosphatase 2C 34 |
| MSTRG.30648 | -2.28367 | down | Abscisic acid 8'-hydroxylase 1 |
| Hca.08G0024360 | 1.308936 | up | Abscisic acid 8'-hydroxylase 1 |
| Hca.06G0040030 | 1.019743 | up | zeaxanthin epoxidase, chloroplastic |
| MSTRG.28014 | 6.641463 | up | putative 9-cis-epoxycarotenoid dioxygenase NCED5 |
| Hca.13G0005170 | 2.019628 | up | 9-cis-epoxycarotenoid dioxygenase NCED3, chloroplastic-like |
| Hca.15G0023070 | 1.407684 | up | protein MARD1-like |
| Hca.07G0037740 | -1.38086 | down | CBL-interacting protein kinase 32 isoform X1 |
| MSTRG.17457 | -2.04247 | down | CBL-interacting protein kinase 18-like |
| Hca.01G0040240 | 1.154215 | up | CBL-interacting protein kinase 23 |
| Hca.13G0014870 | 1.72724 | up | CBL-interacting protein kinase 1 |
| MSTRG.7054 | 1.14316 | up | CBL-interacting protein kinase 18 |
| Hca.13G0023930 | -1.66891 | down | calmodulin-binding transcription activator 5-like isoform X1 |
| Hca.05G0024960 | -5.97126 | down | Calmodulin-binding family protein, putative |
| MSTRG.37189 | -1.2355 | down | calcium/calmodulin-regulated receptor-like kinase 1 |
| MSTRG.20379 | -1.26448 | down | putative calmodulin binding protein |
| MSTRG.4158.1 | 3.148574592 | up | Serine/threonine-protein kinase,SnRK |

DEGs related to drought signal in DvsD-SA

| Gene ID | Log2(Fold Change) | Up/Down | Description |
| --- | --- | --- | --- |
| MSTRG.184 | 1.66698 | up | Abscisic acid receptor PYL5 |
| MSTRG.28014 | -2.27359 | down | putative 9-cis-epoxycarotenoid dioxygenase NCED5 |
