# Supplementary material for: Physiological and Transcriptome Analysis Reveal the Underlying Mechanism of Salicylic Acid-Alleviated Drought Stress in Kenaf (Hibiscus cannabinus L.)
Source: Life (Basel). 2025 Feb 12;15(2):281. doi: 10.3390/life15020281 (PMC11856667; doi:10.3390/life15020281)
Supplement: Supplementary file 1 [file life-15-00281-s001.zip › Table S3.docx]

**Suppl. Table S3** Transcription factors response to drought stress (CKvsD)

| Family | seq_ID | Log2(Fold Change) | Up/Down | Description |
| --- | --- | --- | --- | --- |
| AUX | Hca.03G0041990 | -2.08101 | down | AUXin-responsive protein IAA26 |
|  | Hca.07G0041420 | 1.348561 | up | AUXin-responsive protein IAA27 |
|  | Hca.09G0006170 | 1.638629 | up | auxin-responsive protein IAA26-like |
|  | Hca.08G0020690 | 1.606907 | up | Auxin-responsive protein IAA11 |
|  | MSTRG.19865 | 1.142888 | up | Auxin-responsive protein IAA13 |
|  | Hca.02G0034900 | 1.299469 | up | Auxin-responsive protein IAA16 |
| B3 | Hca.05G0012920 | -8.23158 | down | B3 domain-containing protein |
|  | Hca.06G0040060 | 2.834449 | up | B3 domain-containing protein |
|  | Hca.08G0022960 | -1.67717 | down | B3 domain-containing protein |
|  | Hca.08G0017680 | 3.713877 | up | B3 domain-containing protein |
| bHLH | Hca.08G0021140 | -1.50074 | down | BHLH-MYC and R2R3-MYB Transcription factors N-terminal |
|  | Hca.06G0032320 | -2.52013 | down | BHLH-MYC and R2R3-MYB Transcription factors N-terminal |
|  | MSTRG.9999 | -2.78402 | down | Helix-loop-helix DNA-binding domain |
|  | Hca.08G0021140 | -1.50074 | down | Helix-loop-helix DNA-binding domain |
| BSD | Hca.05G0002570 | 1.165555 | up | BSD domain |
| C2H2 | Hca.05G0000160 | -1.69037 | down | Zinc finger, C2H2 type |
| C3H | Hca.18G0013240 | 1.679776 | up | Zinc finger CCCH domain-containing protein 20 |
|  | MSTRG.28111 | 2.913061 | up | Zinc finger CCCH domain-containing protein 20 |
|  | Hca.08G0002910 | 2.201752 | up | Zinc finger CCCH domain-containing protein 20 |
|  | Hca.06G0040790 | -1.65883 | down | zinc finger CCCH domain-containing protein 41-like |
|  | Hca.07G0039980 | 1.85942 | up | zinc finger CCCH domain-containing protein 23-like |
| CAMTA | MSTRG.30141 | -2.27871 | down | IQ calmodulin-binding motif family protein |
|  | MSTRG.30142 | 2.058959 | up | IQ calmodulin-binding motif family protein, |
|  | Hca.10G0001070 | 1.548555 | up | IQ calmodulin-binding motif family protein, |
| AP2  /ERF | Hca.16G0025780 | -1.82892 | down | ethylene-responsive transcription factor ERF061-like |
|  | Hca.03G0025500 | -1.26617 | down | ethylene-responsive transcription factor ERF070-like |
|  | Hca.05G0014180 | 1.039979 | up | Ethylene-responsive transcription factor ERF060 |
|  | Hca.11G0029690 | 2.109906 | up | ethylene-responsive transcription factor ERF106 |
|  | Hca.12G0005820 | -2.56313 | down | Ethylene-responsive transcription factor 1 |
|  | Hca.10G0000750 | -3.42907 | down | ethylene-responsive transcription factor TINY-like |
|  | MSTRG.26004 | 1.455395 | up | Ethylene-responsive transcription factor 4 |
|  | MSTRG.7081 | 1.6961 | up | Ethylene-responsive transcription factor 4 |
|  | Hca.09G0024100 | 1.040647 | up | ethylene-responsive transcription factor RAP2-3 |
| G2-like | MSTRG.21518 | 1.661119 | up | MYB-like DNA-binding domain |
|  | MSTRG.9682 | 3.705171 | up | MYB-like DNA-binding domain |
|  | Hca.18G0001980 | -1.98919 | down | Myb-like DNA-binding domain |
|  | MSTRG.30511 | 1.649285 | up | Myb-like DNA-binding domain |
| HD-ZIP  MYB | Hca.04G0029170 | 2.831946 | up | Homeobox-leucine zipper protein ATHB-12 |
|  | MSTRG.11671 | 3.516943 | up | Homeobox-leucine zipper protein HOX6 |
|  | MSTRG.3788 | 1.95387 | up | Homeobox-leucine zipper protein HOX19 |
|  | MSTRG.4250 | 3.381523 | up | Homeobox -leucine zipper protein ATHB-40-like |
|  | MSTRG.17954 | 1.117778 | up | Transcription factor MYB44 |
|  | Hca.05G0016340 | 4.123751 | up | MYB-related protein 308 |
|  | MSTRG.8130 | -2.43214 | down | transcription repressor MYB4-like |
|  | MSTRG.3925 | -5.0303 | down | transcription repressor MYB4-like |
|  | Hca.18G0001980 | -1.98919 | down | myb-related protein 308-like |
|  | MSTRG.34637 | -3.84366 | down | Myb-related protein Myb4 |
|  | Hca.18G0001990 | -3.1733 | down | transcription factor MYB74 |
|  | MSTRG.28158 | -5.2164 | down | transcription factor MYB14-like |
|  | MSTRG.31077 | -1.24689 | down | Transcription factor MYB51 |
| NAC | MSTRG.23933 | 4.371028519 | up | NAC domain-containing protein 55 |
|  | MSTRG.3897 | -1.41848363 | down | NAC domain-containing protein 71 |
| NF-Y | Hca.08G0010830 | 4.90068406 | up | nuclear transcription factor Y subunit A-1-like |
|  | Hca.07G0032910 | 1.461669754 | up | Nuclear transcription factor Y subunit A-3 |
|  | MSTRG.26423 | -2.45054811 | down | nuclear transcription factor Y subunit B-3-like |
|  | MSTRG.7394 | 1.035679402 | up | nuclear transcription factor Y subunit B-3-like |
| TCP | Hca.17G0016730 | 1.517819596 | up | transcription factor TCP15-like |
| WRKY | Hca.09G0011890 | -4.04186738 | down | probable WRKY transcription factor 51 |
|  | MSTRG.32326 | -4.21242258 | down | probable WRKY transcription factor 40 |
|  | Hca.09G0003050 | 1.854449121 | up | probable WRKY transcription factor 65 |
|  | Hca.10G0003260 | -5.611444026 | down | probable WRKY transcription factor 40 |
|  | Hca.10G0003250 | -4.683111727 | down | probable WRKY transcription factor 40 |
|  | Hca.02G0015530 | -2.297706595 | down | probable WRKY transcription factor 51 |
|  | Hca.15G0008760 | -1.791103876 | down | probable WRKY transcription factor 53 |
|  | Hca.07G0034360 | -1.434447966 | down | probable WRKY transcription factor 40 |
|  | Hca.05G0023800 | -2.934685308 | down | probable WRKY transcription factor 70 |
|  | Hca.03G0015270 | -2.068701693 | down | probable WRKY transcription factor 53 |

Transcription factors response to drought stress (DvsD-SA)

| Family | seq_ID | Log2(Fold Change) | Up/Down | Description |
| --- | --- | --- | --- | --- |
| AP2/ERF | MSTRG.26004 | -1.38929 | down | Ethylene-responsive transcription factor 4 |
| NF-Y | Hca.08G0010830 | -1.90685 | down | nuclear transcription factor Y subunit A-1-like |
