# Supplementary material for: Physiological and Transcriptome Analysis Reveal the Underlying Mechanism of Salicylic Acid-Alleviated Drought Stress in Kenaf (Hibiscus cannabinus L.)
Source: Life (Basel). 2025 Feb 12;15(2):281. doi: 10.3390/life15020281 (PMC11856667; doi:10.3390/life15020281)
Supplement: Supplementary file 1 [file life-15-00281-s001.zip › Table S4.docx]

**Suppl. Table S4** DEGs invoved in defense responses to drought stress (CK VS D)

| Class | Gene ID | Log2 (Fold Change) | Up/Down | Description |
| --- | --- | --- | --- | --- |
| Reactive oxygen species (ROS) | Hca.01G0019050 | -3.019201982 | down | Superoxide dismutase |
|  | Hca.08G0021980 | -2.302676807 | down | Superoxide dismutase |
|  | Hca.02G0008490 | 2.071478994 | up | peroxidase 17-like |
|  | Hca.02G0008490 | 2.071478994 | up | Peroxidase 17 |
|  | Hca.01G0028490 | -1.699098501 | down | peroxidase 12 |
|  | Hca.09G0019270 | -2.571712344 | down | Peroxidase 12 |
|  | Hca.12G0029510 | -2.002625519 | down | Peroxidase 4 |
|  | Hca.18G0020260 | -3.520419602 | down | peroxidase 43 |
|  | MSTRG.2228 | 1.798835473 | up | Plant peroxidase |
|  | MSTRG.4309 | -3.386221904 | down | Peroxidase 73 |
|  | Hca.04G0003980 | -4.836829115 | down | Plant peroxidase |
|  | Hca.09G0024670 | -2.7930818 | down | peroxidase 29 |
|  | Hca.05G0011060 | -2.690057794 | down | Peroxidase 73 |
|  | Hca.17G0006330 | -1.738943177 | down | glutathione S-transferase U17-like |
|  | Hca.01G0036700 | -3.02853446 | down | glutathione S-transferase U17-like |
|  | Hca.02G0021110 | -1.225757348 | down | glutathione S-transferase DHAR2-like |
| Drought stress responsive proteins | Hca.03G0040950 | 1.877910972 | up | 17.4 kDa class III heat shock protein-like |
|  | MSTRG.20718 | -1.066210456 | down | heat shock cognate protein 80-like |
|  | Hca.01G0007280 | -1.345648504 | down | heat shock cognate protein 80-like |
|  | MSTRG.10429 | -1.172547317 | down | heat shock cognate 70 kDa protein 2-like |
|  | MSTRG.13658 | -2.184786059 | down | Heat shock 22 kDa protein |
|  | Hca.11G0010640 | 1.072225681 | up | Heat shock protein 70 family |
|  | Hca.13G0025590 | 1.89239702 | up | 22.0 kDa class IV heat shock protein-like |
|  | Hca.13G0027480 | 1.081548974 | up | heat shock 70 kDa protein 6 |
|  | Hca.09G0023870 | 2.794567934 | up | late embryogenesis abundant protein Lea5-D |
|  | Hca.15G0023050 | 4.534770242 | up | late embryogenesis abundant protein D-34-like |
|  | Hca.04G0019090 | 3.244622817 | up | Late embryogenesis abundant protein D-11 |
|  | Hca.06G0029500 | 1.561391743 | up | late embryogenesis abundant protein D-34-like |
| Lignin biosynthetic | Hca.06G0013600 | -2.620881357 | down | 4-coumarate--CoA ligase 2-like |
|  | Hca.18G0005230 | -7.220202998 | down | cinnamoyl-CoA reductase 1-like |
|  | Hca.18G0005460 | 1.05911449 | up | cinnamoyl-CoA reductase 1-like |
|  | MSTRG.15294 | 1.337963357 | up | cinnamoyl-CoA reductase 1-like |
|  | Hca.01G0039000 | -1.266516477 | down | Caffeoylshikimate esterase,CSE |
|  | Hca.15G0021770 | 1.326409054 | up | caffeoylshikimate esterase-like |
|  | MSTRG.28325 | -1.313260384 | down | Caffeoyl-CoA O-methyltransferase 1,CCOMT1 |
|  | Hca.06G0003060 | -1.466729906 | down | Caffeoyl-CoA O-methyltransferase,CCOMT |
|  | Hca.07G0020110 | -3.236894089 | down | laccase-5-like |
|  | Hca.08G0012130 | -3.000176933 | down | laccase-7-like |
|  | Hca.13G0013640 | -2.305768448 | down | laccase-17-like |
|  | Hca.06G0007170 | -2.682509381 | down | laccase-17-like |
| Sugar | Hca.01G0027470 | 1.722173588 | up | Aldehyde dehydrogenase family 3 member H1 |
|  | MSTRG.23710 | 3.563789161 | up | Transketolase, pyrimidine binding domain |
|  | Hca.04G0027710 | -1.324990398 | down | Pectinesterase 2 |
|  | MSTRG.19956 | -1.78712912 | down | Endoglucanase 11 |
|  | Hca.15G0008690 | -2.086462575 | down | Endoglucanase 6 |
|  | Hca.01G0009130 | -1.193035887 | down | endoglucanase 10 |
|  | MSTRG.28748 | -2.101919813 | down | endoglucanase 8-like |
|  | Hca.03G0038920 | -1.123988324 | down | endoglucanase 10-like |
|  | Hca.10G0027810 | -1.57960267 | down | endoglucanase 8-like |
|  | Hca.07G0008100 | -1.485948917 | down | endoglucanase 6-like |
|  | Hca.05G0009770 | -1.55960403 | down | UDP-glucose 6-dehydrogenase 2 |
|  | MSTRG.21684 | -1.03577081 | down | UDP-glucose 6-dehydrogenase 1 |
|  | Hca.01G0054520 | -2.925155819 | down | UDP-glucuronate 4-epimerase 3 |
|  | Hca.15G0017310 | -1.815163768 | down | UDP-glucuronate 4-epimerase 3,GAE3 |
|  | Hca.05G0033120 | -2.289559134 | down | probable pectate lyase 13 |
|  | Hca.01G0016290 | -2.683042788 | down | probable pectate lyase 12 |
|  | Hca.04G0015240 | -1.100505976 | down | probable pectate lyase 18 |
|  | Hca.04G0025320 | -1.547063463 | down | probable pectate lyase 20 |
|  | Hca.15G0014400 | -2.324264881 | down | probable pectate lyase 8 |
|  | Hca.04G0008010 | -1.774700327 | down | probable pectate lyase 5 |
|  | Hca.06G0030770 | 1.690606711 | up | mannan endo-1,4-beta-mannosidase 4 |
|  | Hca.07G0013660 | -3.419617844 | down | phosphoglycerate kinase, cytosolic |
|  | Hca.13G0008030 | 1.458020194 | up | alcohol dehydrogenase-like 7 |
|  | Hca.06G0014460 | -1.082182047 | down | alcohol dehydrogenase-like 2 |
|  | Hca.12G0027620 | -1.708056379 | down | UDP-glucuronic acid decarboxylase 5 |
|  | Hca.04G0027610 | -1.753420672 | down | UDP-glucuronic acid decarboxylase 2-like protein |
|  | Hca.05G0002070 | -1.055152304 | down | UDP-glucuronic acid decarboxylase 2 |
|  | MSTRG.36746 | -1.767769082 | down | UDP-glucuronic acid decarboxylase 6 |
|  | Hca.12G0002690 | -2.034045134 | down | endochitinase PR4-like |
|  | Hca.12G0027690 | -2.363544275 | down | chitinase 2-like |
|  | MSTRG.37346 | -1.837423175 | down | Trifunctional UDP-glucose 4,6-dehydratase |
|  | MSTRG.4555 | -2.654385421 | down | Trifunctional UDP-glucose 4,6-dehydratase |
|  | Hca.05G0016800 | -1.207056165 | down | trifunctional UDP-glucose 4,6-dehydratase |
|  | MSTRG.1062 | 1.069779756 | up | beta-amylase 1, chloroplastic-like |
|  | Hca.14G0013700 | 1.204891642 | up | beta-amylase 1, chloroplastic-like |
|  | Hca.11G0012180 | -2.557985786 | down | Raffinose synthase |
|  | Hca.02G0000630 | -1.227455609 | down | Phosphomannomutase |
|  | MSTRG.26656 | -1.326854121 | down | Enoyl-[acyl-carrier-protein] reductase [NADH], chloroplastic |
|  | Hca.08G0019860 | -2.071212982 | down | probable pectinesterase 8 |
|  | MSTRG.34899 | -2.193254865 | down | pectinesterase inhibitor 6 |
|  | Hca.04G0027710 | -1.324990398 | down | pectinesterase-like |
|  | Hca.07G0040570 | -2.485802502 | down | Pectinesterase, catalytic |
|  | Hca.01G0052950 | -1.063492397 | down | probable beta-D-xylosidase 2 |

DEGs invoved in defense responses to drought stress (DvsD-SA)

| Class | Gene ID | Log2 (Fold Change) | Up/Down | Description |
| --- | --- | --- | --- | --- |
| Sugar | Hca.12G0002690 | 1.853909687 | up | endochitinase PR4-like |
|  | MSTRG.37346 | 1.089425183 | up | Trifunctional UDP-glucose 4,6-dehydratase |
