# Supplementary material for: Physiological and Transcriptome Analysis Reveal the Underlying Mechanism of Salicylic Acid-Alleviated Drought Stress in Kenaf (Hibiscus cannabinus L.)
Source: Life (Basel). 2025 Feb 12;15(2):281. doi: 10.3390/life15020281 (PMC11856667; doi:10.3390/life15020281)
Supplement: Supplementary file 1 [file life-15-00281-s001.zip › Table S5.docx]

**Table S5** Confirmation of the expression profiles of selected genes by qRT-PCR

| Gene ID | Protein identity | RNA Seq (FC) | qRT-PCR (FC) |
| --- | --- | --- | --- |
| *Hca.12G0008280* | abscisic acid receptor PYL4-like | 0.030 | 0.67 |
| *MSTRG.37054* | Probable E3 ubiquitin-protein ligase XERICO | 22.28 | 40.50 |
| *MSTRG.1668* | putative protein phosphatase 2C 75 | 5.79 | 3.74 |
| *MSTRG.28014* | putative 9-cis-epoxycarotenoid dioxygenase NCED5 | 99.73 | 36.93 |
| *MSTRG.23933* | NAC domain-containing protein 55 | 20.68 | 13.00 |
| *Hca.05G0024960* | Calmodulin binding family protein, putative | 0.016 | 0.06 |
| *Hca.01G0019050* | Superoxide dismutase | 0.12 | 0.09 |
| *Hca.18G0020260* | peroxidase 43 | 0.087 | 0.38 |
| *Hca.15G0023050* | late embryogenesis abundant protein D-34-like | 23.10 | 4.02 |
| *Hca.04G0019090* | Late embryogenesis abundant protein D-11 | 7.44 | 10.22 |
| *MSTRG.9682* | MYB-like DNA-binding domain | 12.99 | 6.57 |
| *Hca.05G0016340* | MYB-related protein 308 | 17.39 | 6.47 |
