# Supplementary material for: Physiological and Transcriptome Analysis Reveal the Underlying Mechanism of Salicylic Acid-Alleviated Drought Stress in Kenaf (Hibiscus cannabinus L.)
Source: Life (Basel). 2025 Feb 12;15(2):281. doi: 10.3390/life15020281 (PMC11856667; doi:10.3390/life15020281)
Supplement: Supplementary file 1 [file life-15-00281-s001.zip › Table S7.docx]

**Table S7.**

**Primers of qRT-PCR**

| **Primer name** | **Sequence (5’ → 3’)** |
| --- | --- |
| *Hca.12G0008280*-F | GCTCCGTGCTCCTTGCC |
| *Hca.12G0008280*-R | TGTTGCTCCGCCGTCGTA |
| *MSTRG.37054*-F | TCCATCGGCTCGGCTCT |
| *MSTRG.37054*-R | CCTTATGGAAAAGATGGTCGC |
| *MSTRG.1668*-F | TCAAGCGTTCATTGGGGC |
| *MSTRG.1668*-R | CTATTTCTCTTATGGCTGGTTTGG |
| *MSTRG.28014*-F | ACAGTCCCCGCTTTACCCTC |
| *MSTRG.28014*-R | GCCATAGCCGCAACTCTTT |
| *Hca.05G0024960*-F | TCGCATTGGTTGTGTGAGGG |
| *Hca.05G0024960*-R | CAGTCCGAGGTAGGCAAGTGTAG |
| *Hca.01G0019050*-F | GAGACCCTCACTCGCCATTG |
| *Hca.01G0019050*-R | TTCTCTAACAAAGACGACGGTAAAT |
| *Hca.18G0020260*-F | AATGGGTTTTGCTTTCTTGTTTC |
| *Hca.18G0020260*-R | CTGACTCGGCATCTGGGC |
| *Hca.15G0023050*-F | CACCGTAACTAAGTCCACCGC |
| *Hca.15G0023050*-R | CCCCACAACCTTTCTCATCAA |
| *Hca.04G0019090*-F | GGGCACAAAGAGCAGCAAAC |
| *Hca.04G0019090*-R | TCACACCCTTCTTCTCCCCA |
| *MSTRG.9682*-F | ACGAACTCGGCTGAGACGG |
| *MSTRG.9682*-R | AGTCCAAACCCTGGCGAAG |
| *Hca.05G0016340*-F | AAACAGTCGGTGTCGCCATT |
| *Hca.05G0016340*-R | TGGAAGCCCAATGGAGAGTT |
| *MSTRG.23933*-F | TGGATTGGGCGAACCTTG |
| *MSTRG.23933*-R | TCTCTACCTCCTCTTCCACCGA |
| *HcSABP2*-F | CTGTTTTCTTGACCGCTTTCAT |
| *HcSABP2*-R | GCTTCTTTTGGTGTTCTTTCCC |
| *Actin3*-F | GTGAGGATATTCAACCCCTTGTCT |
| *Actin3*-R | CATCTTTCTGTCCCATACCAACC |

**Primers of VIGS**

| **Primer name** | **Sequence (5’ → 3’)** |
| --- | --- |
| TRV-*HcSABP2*-F | GATTCTGTGAGTAAGGTTACCGAATTCAAGGTTATTCTCGTTGGGCA |
| TRV-*HcSABP2*-R | CCCCATGGAGGCCTTCTAGAGAATTCCTGCCTTGGACAAATCGG |
