# Supplementary figures and images for: Physiological and Transcriptome Analysis Reveal the Underlying Mechanism of Salicylic Acid-Alleviated Drought Stress in Kenaf (Hibiscus cannabinus L.)
Source: Life (Basel). 2025 Feb 12;15(2):281. doi: 10.3390/life15020281 (PMC11856667; doi:10.3390/life15020281)

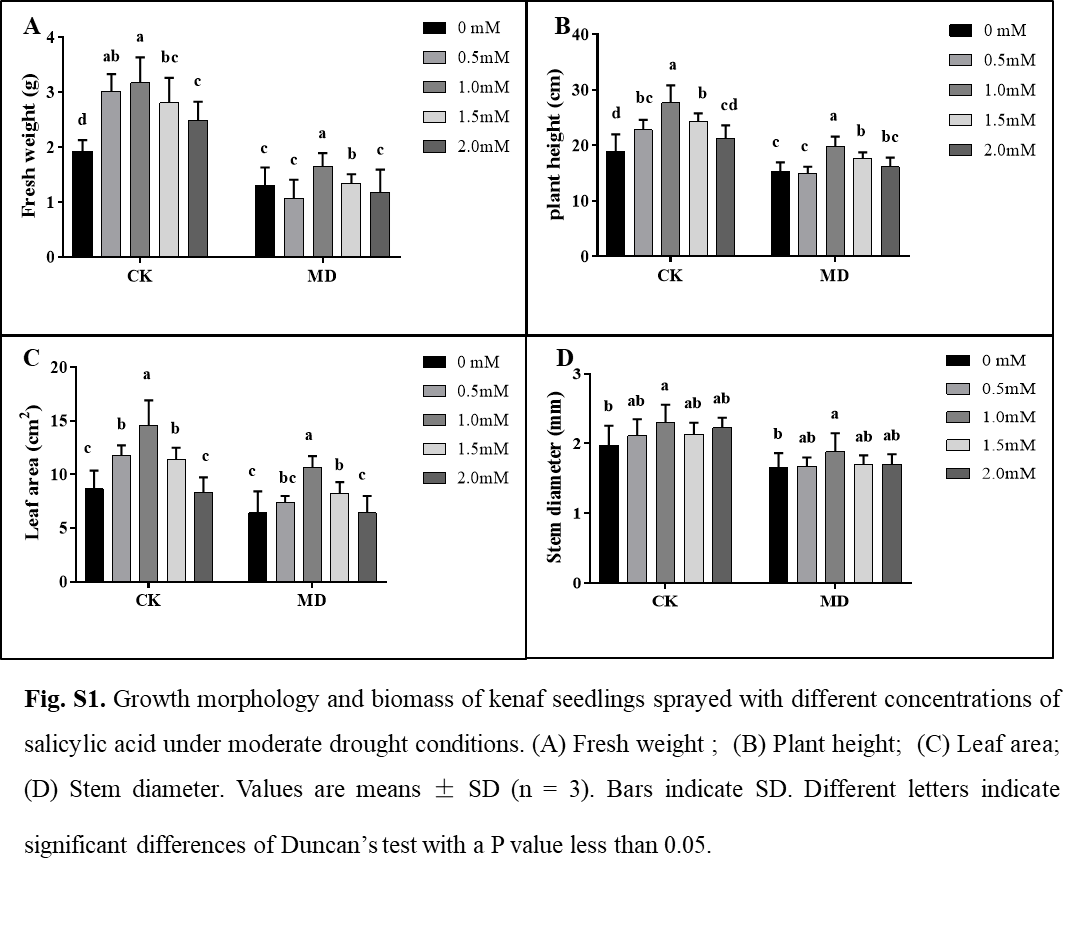

Supplement: Supplementary file 1 [file life-15-00281-s001.zip › Fig. S1.docx]

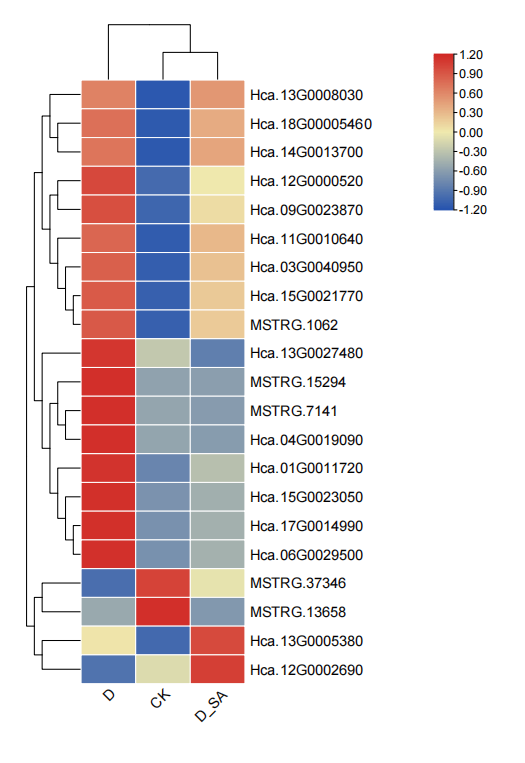


Fig. S2. Heat map of genes associated with defense response to drought stress

Supplement: Supplementary file 1 [file life-15-00281-s001.zip › Fig. S2.docx]

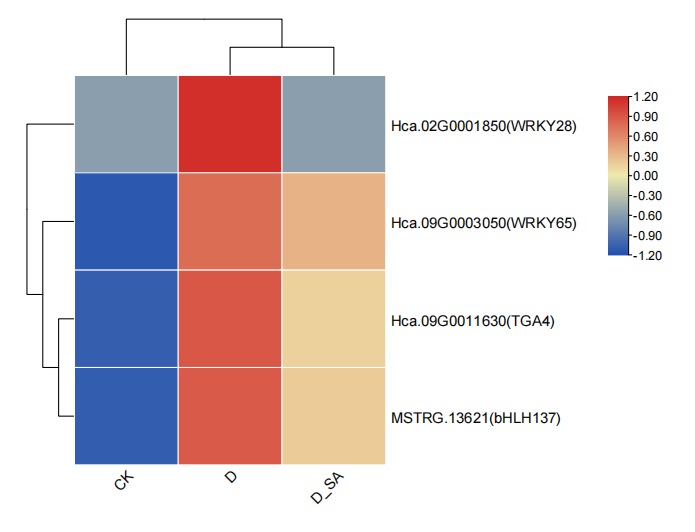


Fig. S4. Heat map of salicylic acid-related genes

Supplement: Supplementary file 1 [file life-15-00281-s001.zip › Fig. S4.docx]
